# Mechanochemical P-derivatization of 1,3,5-Triaza-7-Phosphaadamantane (PTA) and Silver-Based Coordination Polymers Obtained from the Resulting Phosphabetaines

**DOI:** 10.3390/molecules25225352

**Published:** 2020-11-16

**Authors:** Antal Udvardy, Csenge Tamara Szolnoki, Réka Gombos, Gábor Papp, Éva Kováts, Ferenc Joó, Ágnes Kathó

**Affiliations:** 1 Department of Physical Chemistry, University of Debrecen, P.O. Box 400, H-4002 Debrecen, Hungary; szolnoki.csenge@science.unideb.hu (C.T.S.); gombos.reka@science.unideb.hu (R.G.); papp.gabor@science.unideb.hu (G.P.); katho.agnes@science.unideb.hu (Á.K.); 2 Doctoral School of Chemistry, University of Debrecen, P.O. Box 400, H-4002 Debrecen, Hungary; 3 Institute for Solid State Physics and Optics, Wigner Research Centre for Physics, Konkoly Thege Miklós u. 29-33, H-1121 Budapest, Hungary; kovats.eva@wigner.mta.hu; 4 MTA-DE Redox and Homogeneous Catalytic Reaction Mechanisms Research Group, P.O. Box 400, H-4002 Debrecen, Hungary

**Keywords:** antimicrobial activity, ball mill, coordination polymer, mechanochemistry, phosphonium salt, silver, 1,3,5-Triaza-7-phosphaadamantane (PTA)

## Abstract

We have described earlier that in aqueous solutions, the reaction of 1,3,5-triaza-7-phosphaadamantane (PTA) with maleic acid yielded a phosphonium-alkanoate zwitterion. The same reaction with 2-methylmaleic acid (citraconic acid) proceeded much slower. It is reported here, that in the case of glutaconic and itaconic acids (constitutional isomers of citraconic acid), formation of the corresponding phosphabetaines requires significantly shorter reaction times. The new phosphabetaines were isolated and characterized by elemental analysis, multinuclear NMR spectroscopy and ESI-MS spectrometry. Furthermore, their molecular structures in the solid state were determined by single crystal X-ray diffraction (SC-XRD). Synthesis of the phosphabetaines from PTA and unsaturated dicarboxylic acids was also carried out mechanochemically with the use of a planetary ball mill, and the characteristics of the syntheses in solvent and under solvent-free conditions were compared. In aqueous solutions, the reaction of the new phosphabetaines with Ag(CF_3_SO_3_) yielded Ag(I)-based coordination polymers. According to the SC-XRD results, in these polymers the Ag(I)-ion coordinates to the N and O donor atoms of the ligands; however, Ag(I)-Ag(I) interactions were also identified. The Ag(I)-based coordination polymer (**CP1.2**) formed with the glutaconyl derivative of PTA (**1**) showed considerable antimicrobial activity against both Gram-negative and Gram-positive bacteria and yeast strains

## 1. Introduction

Most chemical reactions proceed in solution. The use of solvents allows the reactions to take place between molecularly dispersed reactants, help the agitation and transport of the reaction mixture, or to maintain a proper reaction temperature (i.e., at reflux conditions), and facilitate the isolation of the products by filtration, extraction, etc. [1]. Unfortunately, in many cases the suitable solvents are expensive volatile, flammable, and toxic organic compounds which pose an environmental threat. In green syntheses, however, the use of the minimum quantities, if any, of auxiliaries, such as solvents, are required, or at least the dangerous organic solvents should be replaced by less harmful alternatives, such as e.g., water [2,3]. Even in the case of water there is a possibility to contaminate the environment with spent solvent. Furthermore, product isolation by distilling the aqueous solvent is highly energy consuming, which at the far end is also a burden on the environment. 

According to a well-known saying ‘the best solvent is no solvent’, and recent research into mechanochemistry has proven that, indeed, in many cases it is possible to perform reactions with the same or better efficiency in the solid state than in solution [4,5,6,7]. In recognition of its far-reaching advances, mechanochemistry was identified by IUPAC as one of the 10 world-changing technologies [8].

The number of solid-phase reactions have increased dramatically in the last few years. The mechanical energy of milling allowed successful realization of the most diverse chemical reactions [9,10], such as Pd-catalyzed C-C couplings [11,12,13], hydrogenation [14,15], halogenation [16], oxidation [17], N-alkylation [18], P-alkylation of PPh_3_ [19], polymerization [20], and click-reactions [21], to name a few. Complexes of various transition metals (Cu [22], Ag [23,24], Au [23], Pd [25], Ru [26]) have also been synthetized in suitable mills; such syntheses have been reviewed [27]. Recently, we reported the mechanochemical synthesis of Rh(I)-*bis*-NHC (NHC = N-heterocyclic carbene) complexes [28]. In most cases, the mechanochemical reactions are performed in planetary or vibrational ball mills (pbm and vbm, respectively), and the reaction conditions can be finely tuned by proper setting of the milling parameters such as rotation speed, milling time, the material and size of the milling jar and the balls.

**Figure 1:**
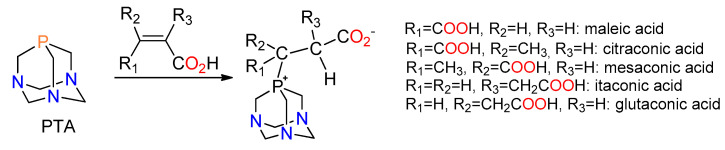
PTA and PTA-derived phosphonium salts.

The water-soluble 1,3,5-triaza-7-phosphaadamantane (PTA) (Figure 1) is a frequently used tertiary phosphine ligand of several precious metal-based catalysts applied in aqueous or aqueous-organic biphasic reactions [29,30,31]. PTA is also able to form coordination polymers due to the presence of several donor atoms in the molecule [29,30,31]. A case in the point is the formation of a two-dimensional coordination network formed in the reaction of PTA and AgNO_3_ [32]. In the presence of dicarboxylic acids, this network is significantly modified as a result of coordination of the carboxylate groups to Ag(I). Such Ag-based coordination polymers displayed antimicrobial activity [33,34].

It is described in the literature, that in solution, PTA is N-alkylated by alkyl halides. We have successfully performed the reactions of PTA and alkyl halides in a planetary ball mill. The yields of high purity, isolated N-alkyl-PTA products were higher than those obtained with the use of organic solvents [35].

In contrast to the easy formation of N-alkylated PTA derivatives, P-alkylation of PTA is rarely observed. One of such products is 7-(2-carboxy-ethyl)-1,3,5-triaza-7-(phosphoniatricyclo)[3.3.1.1^3,7^]- decane [36,37] detected as an intermediate in the PTA-catalyzed Morita–Baylis–Hillman reaction between aldehydes and acrylic acid esters. We have found, that the direct reaction of maleic, fumaric, citraconic, and mesaconic acids also led to formation of P-alkylated PTA-derivatives [38], albeit the reactions with fumaric, citraconic, and mesaconic acids were slow and/or provided low yields. Due to P-alkylation, the resulting phosphabetaines no longer possess a potent P(III) donor atom; however, in addition to the three N-donor atoms, they also have two carboxylate functions (Figure 1).

We conceived that the PTA-derived phosphabetaines may form complex coordination polymers with silver(I) due to the simultaneous presence of three N donor atoms and two carboxylate groups in the same molecule. With that aim in mind, PTA was P-alkylated with the further two isomers of C5 unsaturated dicarboxylic acids, namely *trans*-pent-2-endioic (glutaconic) acid and 2-methylene-butanedioic (itaconic) acid, which yielded the phosphabetaines **1** and **2**, respectively. (Figure 1) The syntheses were carried out both in aqueous solution and in the solid state in a ball mill. **1** and **2** were characterized by elementary analyses and spectroscopic methods, and by single crystal X-ray diffraction (SC-XRD) measurements. Finally, the new phosphabetaines were reacted in aqueous solution with Ag(CF_3_SO_3_) which, indeed, led to formation of coordination polymers (CP-s). The solid state structures of the latter CP-s were studied in detail by SC-XRD measurements. In addition, the antimicrobial activity of the polymers was also determined against bacterial and yeast species. Results of these studies are described below.

## 2. Results and Discussions

### 2.1. Synthesis of the Phosphabetaines 1 and 2

**Figure 2:**
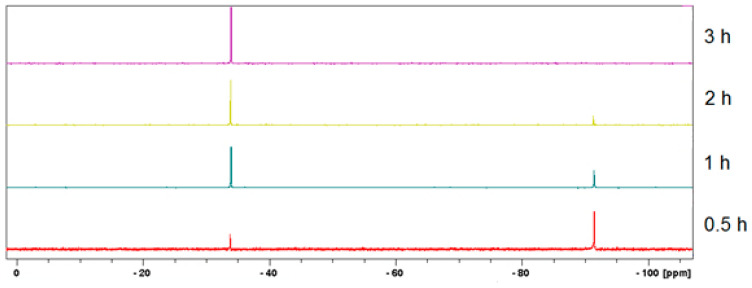
Evolution in time of the ^31^P NMR spectra of aqueous reaction mixtures containing equivalent amounts of PTA and *trans*-glutaconic acid. *Conditions*: PTA (157 mg, 1.0 mmol), *trans*-glutaconic acid (130 mg, 1.0 mmol) in 2.5 mL water, *T* = 70 °C.

**Figure 3:**
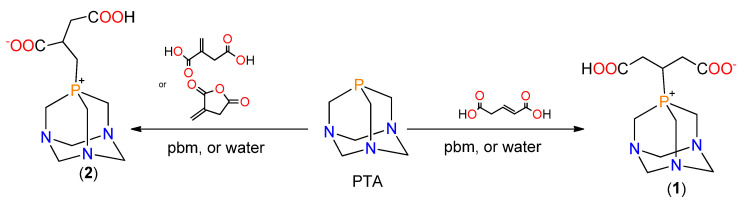
Synthesis of **1** and **2** in water or in a planetary ball mill (pbm).

We have described earlier, that in an equimolar aqueous solution of PTA and fumaric acid at 25 °C, the P-alkyl derivative, 3-carboxy-2-(1,3,5-triaza-7-phosphoniatricyclo[3.3.1.1^3,7^]dec-7-yl)-propanoate (**3**) is formed with a yield of 8.8% (determined by ^31^P NMR spectroscopy) in 24 h. After 8 days the NMR yield was still only 16.4%, together with 4.7% PTA-oxide [38]. In contrast, under identical conditions, *trans*-glutaconic acid reacted substantially faster, so much that after 75 h the ^31^P NMR spectrum of the reaction mixture showed only the presence of the corresponding P-alkylated derivative, 4-carboxy-3-(1,3,5-triaza-7-phosphoniatricyclo-[3.3.1.1^3,7^]dec-7-yl)butanoate (**1**); δ = –34.0 ppm (s). At 70 °C, the reaction went to completion in only 3 h time (Figure 2 and Figure 3).

After evaporation of the solvent at reduced pressure, the remaining solid was washed with chloroform, leading to isolation of **1** with a yield of 87%.

In the HR-MS spectrum of **1** the most intense peak was observed at *m/z* = 288.1107 ([M + H]^+^), however the peak belonging to the dimer [2M + H]^+^ also appeared at *m/z* = 575.2144 (Figure S5). The doublets in the ^13^C{^1^H} NMR spectrum of **1** at 28.26 (^1^*J*_PC_ = 34 Hz) and 48.60 ppm (^1^*J*_PC_ = 34 Hz) are characteristic for the P^+^*C*H-(CH_2_)_2_ and P^+^–*C*H_2_–N carbon atoms [39].

The reaction of itaconic acid and PTA was complete in 54 h at room temperature, i.e., it took place somewhat faster than the alkylation with glutaconic acid. At *T* = 70 °C, the ^31^P NMR signal of (PTA-H)^+^ was hardly detectable after 2 h, instead the resonances of 3-carboxy-2-(1,3,5-triaza-7-phosphoniatricyclo[3.3.1.1^3,7^]dec-7-yl-methyl)propanoate (**2**) grew in at *δ* = −41.6 ppm (s) (Figure S1). The product phosphonium salt was isolated with 71% yield. Reaction of PTA and itaconic anhydride furnished the same product (**2**) with the same yield. 

In the ESI-MS spectrum both the parent molecular ion ([M + H]^+^; *m/z* = 288.1108) and its dimer ([2M + H]^+^; *m/z* = 575.2145) were detected (Figure S9). Addition of the phosphine onto the exo methylene group of itaconic acid is shown by the resonances at 24.0 ppm (d) (P^+^–*C*H_2_–CH) with ^1^*J*_PC_ = 39 Hz, and at 37.12 ppm (d) (P^+^–CH_2_–*C*H) with ^2^*J*_PC_ = 14 Hz. The P^+^–*C*H_2_–N carbon atoms of PTA-moiety resonate at 49.15 ppm (d) with ^1^*J*_PC_ = 39 Hz.

Similar to the previously studied phosphabetaines, **1** and **2** proved stable in air and in aqueous solution, too; their ^31^P NMR spectra remained unchanged after 8 h heating at *T* = 80 °C. In a 5% aqueous H_2_O_2_ solution at room temperature, no phosphine oxide was detected in 8 h. In contrast to the phosphonium salts obtained from sulfonated triphenylphoshines [40], **1** and **2** did not show signs of decomposition in 1 M aqueous NaOH even at elevated temperatures.

Altogether, the reactivity of unsaturated dicarboxylic acids towards PTA in water showed the following order: maleic>>itaconic>*trans*-glutaconic>citraconic>>fumaric>mesaconic acid. 

This is in agreement with the reported order of the reaction rate of maleic and fumaric acid with monosulfonated triphenylphosphine, *m*tppms [41].

The same relative reactivities were observed in solid state reactions of PTA and unsaturated dicarboxylic acids in the planetary ball mill. When PTA was milled together with fumaric acid, or 2-methylfumaric (mesaconic) acid, no reactions were observed in 8 h. The reaction of *cis*-2-methylmaleic (citraconic) acid was very slow; ^31^P NMR of an aqueous solution of the white powder obtained in 8 h showed only 5% conversion to the corresponding phosphabetaine (**4**). In contrast, the solid state reactions of PTA with glutaconic, itaconic, and maleic acids showed 100% conversions already in 4 h. The isolated yields of phosphabetaines **1**–**3**, both from the aqueous solution syntheses and the ball mill reactions are shown Table 1.

It can be seen from Table 1, that under reasonable reaction times (2–4 h) all the studied dicarboxylic acids, except citraconic acid, yielded the expected phosphabetaines with 100% conversion both in solution and in solid state. The reaction of maleic acid and PTA was outstandingly fast leading to full conversion already at 1 °C in 3 h. It is noteworthy that both methods avoid the use of organic solvents (the products are washed off the balls and the walls of the milling jar with a minimum amount of water). Altogether, the mechanochemical synthesis proved a suitable and convenient method of the studied phosphabetaines. The low reactivity of citraconic acid deserves special mention. It was found earlier that at room temperature citraconic acid and PTA yielded the corresponding phosphabetaine with 24% yield in aqueous solution in 9 months (40% yield was observed in 48 h at 70 °C) [38]. With reference to the decomposition of citraconic acid around 90 °C upon attempted melting [42], it may be suggested, that the low yields obtained in solution synthesis at elevated temperature [38], or in the ball mill may be due to decomposition of this diacid under the reaction conditions. We milled citraconic acid alone and also together with an equimolar amount of PTA for 4 h, however, ^1^H and ^13^C NMR spectra of the resulting powders did not show any sign of decomposition products. 

### 2.2. Structural Characterization of the Phosphabetaines 1 and 2

**Figure 4:**
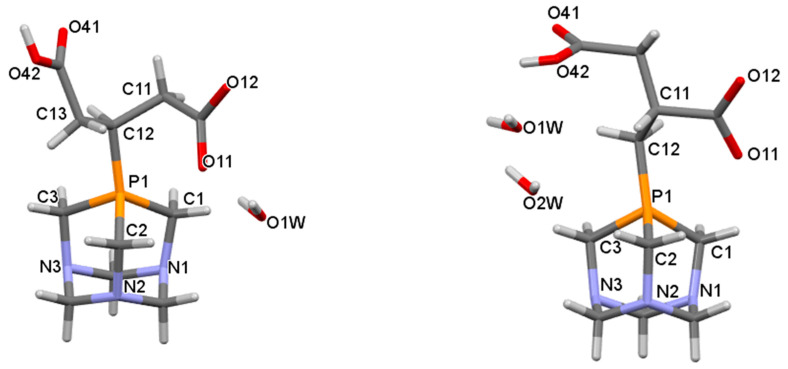
Capped stick representation of **1** × H_2_O (**left**) and **2** × 2H_2_O (**right**) with partial atom numbering. Selected bond lengths and angles are shown in Table S2.

The structure of phosphabetaines **1** and **2** were determined by single crystal X-ray diffraction measurements (Figure 4). Crystals were obtained from aqueous solutions of **1** and **2** which were layered with 2-propanol and stored at 5 °C.

**1** crystallizes in the monoclinic *P*2_1_/*c* space group and its asymmetric unit contains one phosphabetaine molecule and one water, while crystals of **2** belong to the triclinic space group with one zwitterion and two water molecules in the asymmetric unit. In neither case do the lattices contain 2-propanol. ORTEP presentations can be found on Figures S14 and S17.

The structural effects of P-alkylation are obvious (Table S2). The bond distances 1.823(2) Å (P1–C12; **1**) and 1.788(6) Å (P1–C12; **2**) clearly refer to P^+^–C(sp^3^)-type bonds. PTA has a fairly rigid adamantane-type cage structure, and therefore –in general– the bond lengths and angles in **1** and **2** do not differ largely from those in PTA. Still, the C1–P–C2, C2–P–C3, C1–P–C3 bond angles are considerably larger, 103.26(11)°–100.2(3)° than the respective angle, 96.06°, in PTA. Furthermore, and in agreement to earlier literature reports [43], the P1–C1, P1–C2, and P1–C3 bond lengths were found shorter in both phosphabetaines, **1** and **2**, than in PTA.

In the free acids the distances of the olefinic carbon atoms are 1.319 Å[44], and 1.323 Å[45]. In contrast, in **1** the C11–C12 distance is 1.530(3) Å and (C12–C13) is 1.532(3) Å, showing that the respective carbon atoms are C(sp^3^) hybridized. The same is true for the exomethylene C11 carbon and the neighboring C12 in **2**, with the C12–C11 distance being 1.525(8) Å.

The carboxylate groups closer to the positively charged phosphorus are deprotonated in both **1** and **2**, while the distant oxygens are protonated (in **1**: P1–O11 = 2.756(2) Å and in **2** P1–O11 = 2.789(5) Å).

**Figure 5:**
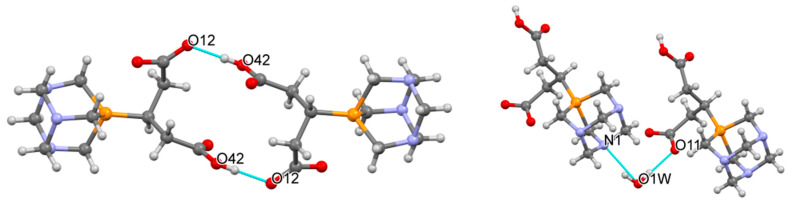
Typical view of **1** in its crystal structure. (**Left**): H-bonds between PTA units, (**Right**): H-bonds between PTA and H_2_O molecules. (O42–H...O12^(ii)^ = 2.571(3) Å, O1W–H...O11 = 2.781(3) Å, O1W–H...N1^(i)^ = 2.889(3) Å [Symmetry codes: (i) x,y,−1 + z, (ii) 1 − x,−y,z]).

**Figure 6:**
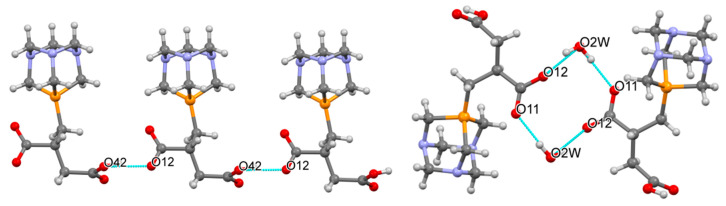
Partial packing view of **2.** (**Left**): Hydrogen bonds between the PTA units. (**Right**): The connection between PTA units via H_2_O molecules (Bond lengths (Å): O42–H...O12^(ii)^ = 2.515(7), O2W–H...O12^(iii)^ = 2.806(8), O2W–H...O11^(ii)^ = 2.725(8), [Symmetry codes: (ii) 1 + x,y,–1 + z (iii) 2 − x,1 − y,1 − z]).

Packing diagrams of **1** and **2** show layered structures (Figure 5 and Figure 6) In the crystals, the polar units (carboxylate groups) turn towards each other and form layers between the layers of apolar groups (PTA) held together by extended hydrogen-bond networks.

In the crystals of **1**, strong hydrogen bonds are formed (O42–H...O12^(ii)^ = 2.571(3) Å) between the protonated oxygen of one phosphabetaine and the deprotonated carboxylate oxygen atom of the other phosphonium salt; between one of the N atoms and a lattice water molecule (O1W–H...N1^(i)^ = 2.889(3) Å, Symmetry code: x,y,–1 + z); and between one of the carboxylate oxygens and a lattice water (O1W–H...O11 = 2.781(3) Å). In addition, there are weak hydrogen bonds between the phosphabetaine chains (Figures S15 and S16, Table S3).

In addition to the zwitterion, there are two water molecules in the crystal lattice of **2**. The long distance arrangement of the molecules shows an extended hydrogen bond network. Importantly, the water molecules form solvate channels within the lattice (Figure 6, Figures S18 and S19, Table S4). Similar to **1**, the strongest hydrogen bonds (O42–H...O12^(ii)^ = 2.515(7) Å) can be observed between the protonated oxygen of one phosphabetaine and the deprotonated carboxylate oxygen atom of the other. The lattice is stabilized by further hydrogen bonds with participation of water oxygens (O2W–H...O12^(iii)^ = 2.806(8) Å, O2W–H...O11^(ii)^ = 2.725(8) Å). In addition, weak C-H…O interactions can also be observed in the crystals. Details can be found in the Supporting Material.

### 2.3. Structure of the Silver-Based Coordination Polymers

**Scheme 1:**
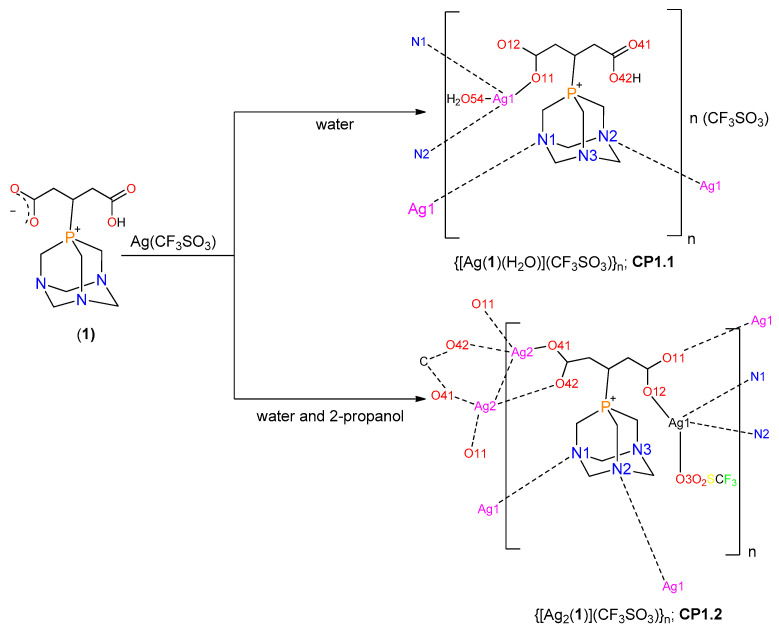
Synthesis of the coordination polymers CP1.1 and CP1.2.

When aqueous solutions of Ag(CF_3_SO_3_) and the phosphabetaine **1** (obtained in the reaction of glutaconic acid and PTA) were mixed and stored at 5 °C, colorless crystals of the coordination polymer **CP1.1** appeared in two weeks (Scheme 1). Upon layering organic solvents (acetone, ethyl alcohol, or 2-propanol) onto the solution, another coordination polymer, **CP1.2** was obtained reproducibly.

An important difference between the two polymers is in that while **CP1.1** (especially in solution) is light sensitive, **CP1.2** is not decomposed by light. Both compounds are insoluble in organic solvents such as alcohols and acetone, and even in DMSO. **CP1.1** dissolves in water, however, its solubility is low. In contrast, **CP.1.2** is well soluble in water. Therefore, solutions for further studies were prepared in water.

In relation to **1**, the ^31^P chemical shifts changed only slightly upon the addition of Ag(CF_3_SO_3_); *δ* = –33.78 (**CP1.1**) and –33.23 (**CP1.2**) ppm (Figure S10 and S11). Similarly, the ^1^H NMR spectra also showed hardly any alterations (Figure S12). Positive ion mode HR ESI-MS displayed peak at *m/z* 288.1108 (**CP1.2**, [M + H]^+^); these values are characteristic for the free phosphabetains. It may be assumed that disruption of the polymers happens during ionization, however, the NMR results strongly suggest that the polymers fall apart already in water. Nevertheless, the good water-solubility of **CP1.2**, and its profound stability to visible light (sunlight) irradiation contrast strikingly to the behavior of **CP1.2** and **CP.2** (see later).

**Figure 7:**
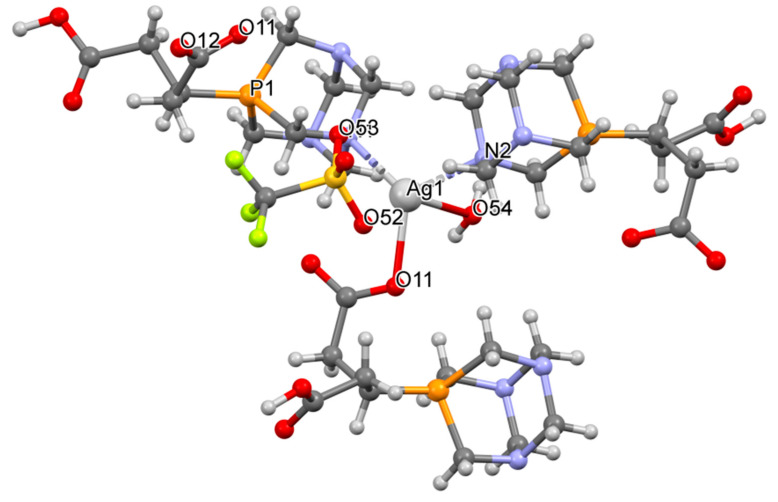
Partial packing view of **CP1.1** Selected bond lengths (Å): Ag1–O11 = 2.294(3), Ag1–N2^(i)^ = 2.439(3), Ag1–N1^(ii)^ = 2.465(3), Ag1–O54 = 2.691(4), P1-O11 = 2.798(3), P1–C12 = 1.818(4), Ag1–O12 = 2.963(3), weak interactions: Ag1–O53^(iii)^ = 3.248(6), Ag1–O52^(iii)^ = 3.166(6) [Symmetry codes: (i) −x, 1 − y,1 − z, (ii) x,3/2 − y,1/2 + z (iii) x,3/2 − y,1/2 + z (iv) 1 − x,−1/2 + y,3/2 − z, (v) –x,2 − y,1 − z].

**CP1.1** is a coordination polymer that crystallizes in *P*2_1_/*c* space group. The crystal structure is built from closed packed 2D polymer sheets separated by triflate ions (Figure 7 and Figures S23). Beside one Ag^+^ ion, the asymmetric unit of the crystal contains one phosphabetaine (O42 is protonated), a water molecule and a triflate ion (Figure S20). The long range order in the crystal shows that each silver ion coordinates to three phosphonium salts; one of them is coordinated through its carboxylate group, while the others are connected with their nitrogen atoms (Figures S21 and S22). A four-coordinated, trigonal pyramidal symmetry around the Ag^+^ (τ_4_ = 0.85, according Houser) [46] is formed by the coordination of a water molecule.

The crystal lattice is stabilized by strong hydrogen bonds between the carboxyl groups of the neighboring polymer sheets (shown by the distances O42–H42...O12^(iv)^ = 2.583(5) Å, O12–H12...O41 = 2.706(5) Å). The coordinated water molecules and triflate ions are also fastened by H-bonds (O54–H54A...O53 = 2.840(6) Å, O54–H54B...O51^(v)^ = 2.893(6) Å). Furthermore, weak C–H…O interactions can be also observed (See Table S5).

**Figure 8:**
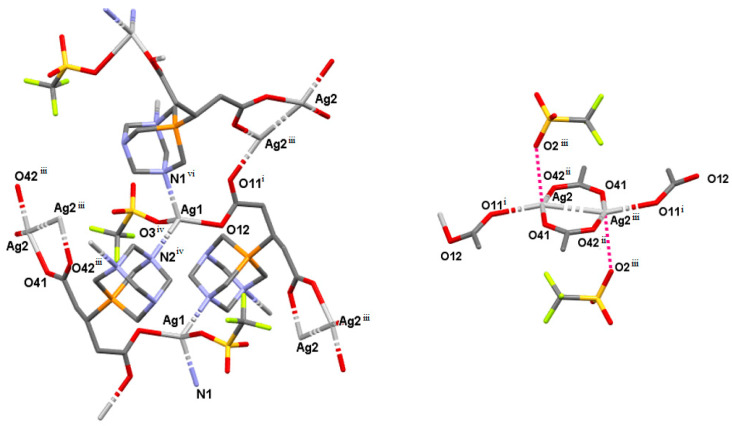
Partial packing view of **CP1.2** with selected bond lengths (**left**). Geometry of Ag2…Ag2 junctions (**right**). Hydrogen atoms are omitted for the clarity. (Bond lengths (Å): Ag1–O12 = 2.282(3), Ag1–O3^(iv)^ = 2.641(6), Ag1–N2^(iv)^ = 2.445(3), Ag2–N1^(vi)^ = 2.420(3), Ag2–Ag2(^iii^) = 2.8987(6), P1–C12 = 1.832(4), P1–O12 = 2.873(3), P1–O42 = 2.858(3), Ag2–O41 = 2.176(5), Ag2–O42^(iii)^ = 2.217(3)Å). Ag2 – O11^(i)^ = 2.474(4), Ag2–O2^(iii)^ = 2.781(5), [Symmetry code: (i) 1/2 – x,–1/2 + y,3/2 – z, (iii) –x,1 – y,1 – z, (iv) 1 – x,1 – y,1 – z, (vi) 1/2 + x,3/2 – y,1/2 + z]).

Based on the solid state structure of **CP1.2**, the compound is a 3D coordination polymer (Figure 8), crystallized in the monoclinic *P*2_1_/*n* space group. Besides two Ag^+^ ions, the asymmetric unit of the unit cell contains one triflate ion and one deprotonated phosphonium salt (Figures S24 and S25). The coordination environments of the two silver ions are different; the coordination polyhedron around Ag1 is a distorted tetrahedron, while that around Ag2 has a square planar geometry.

The four connected dinuclear Ag2 nodes of the complex have special structure. Two Ag2 ions are doubly bridged by the carboxylate groups of two neighboring phosphabetaines (Figure 8). The formed eight-membered planar ring contains two opposite silver ions (rhombic Ag_2_O_2_ units). Such kind of ring structures are common for metal carboxylate complexes, contrarily, in silver complexes there are only six examples of similar structural motif in the CSD database [47]. In this junction, the Ag2…Ag2^(iii)^ distance is 2.8987(6) Å, which is close to that in metallic silver (2.89 Å), moreover, it is substantially shorter than the sum of van der Waals radii of two Ag(I) ions (3.44 Å) [48]. The close interaction of the two silver ions in the nodes is due to the forcing conditions created by the bidentate coordination of the two carboxylate groups.

**Scheme 2:**
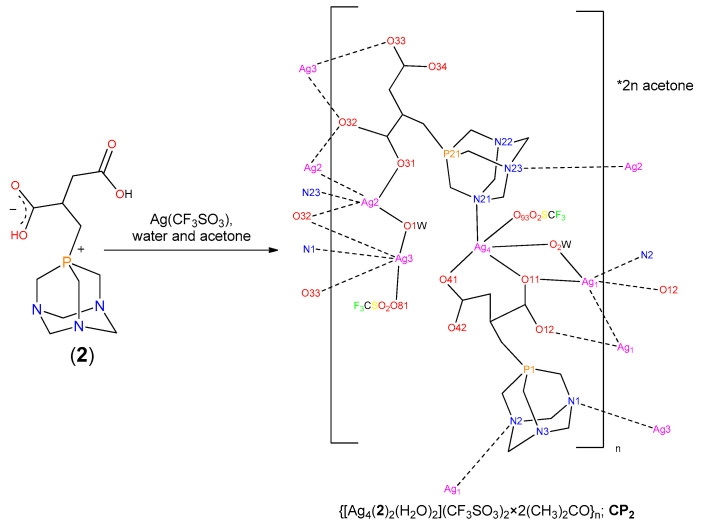
Synthesis of the coordination polymers CP2.

**Figure 9:**
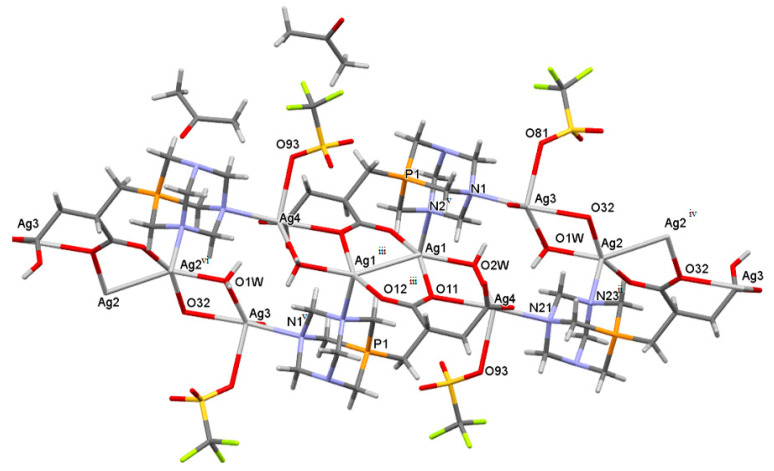
Partial packing view of **CP2**. Selected bond lengths (Å): Ag1–O2W = 2.383(4), Ag1–O11 = 2.319(5), Ag1–Ag1^(iii)^ = 2.8315(6), Ag1–O12^(iii)^ = 2.253(5), Ag1–N2^(v)^ = 2.480(6), Ag2–O1W = 2.377(4), Ag2–O31 = 2.241(5), Ag2–O32 = 2.301(5), Ag2–N23^(ii)^ = 2.493(6), Ag2–Ag2^(iv)^ = 2.8310(6), Ag3–O1W = 2.357(6), Ag3–O32 = 2.668(4), Ag3–O33 = 2.342(6), Ag3–O81 = 2.584(3), Ag3–N1^(v)^ = 2.458(6), Ag3–O42 = 2.672(4), Ag4–O2W = 2.351(6), Ag4–O11 = 2.672(4), Ag4–O41 = 2.343(6), Ag4–O93 = 2.609(9), Ag4–N21 = 2.449(6), Ag1–Ag4 = 3.651, Ag2–Ag3 = 3.641, [Symmetry codes: (ii) 1 + x,y,z, (iii) –x,1 – y, – z, (iv) 1 – x,1 – y,–1-z (v) 1 – x,1 – y,–z ].

Phospabetaine **2** (from PTA and itaconic acid) was also reacted with Ag(CF_3_SO_3_) in aqueous solution (Scheme 2). Layering the reaction mixture with acetone led to formation of light sensitive colorless crystals (**CP2**) together with a white powder and metallic silver. Unfortunately, the efforts to collect sufficient amounts of **CP2** for spectroscopic analysis were not successful, and the compound was characterized only by single-crystal X-ray diffraction (Figure 9).

In contrast to the former structures, **CP2** crystallizes in the low symmetry triclinic *P*–1 space-group as a 2D coordination polymer creating a layered structure (Figure 9). In the asymmetric unit, it contains two molecules of deprotonated phosphabetaines, four Ag(I) ions (each in different environments), two triflates and two-two molecules of acetone and water, each (Figure S26). This leads to the empirical formula of [Ag_4_(**2**)_2_(H_2_O)_2_](CF_3_SO_3_)_2_×2(CH_3_)_2_CO.

The four core nodes are connected through phosphabetaine bridges. In each node, all silver(I) ions are surrounded by distorted square pyramidal coordination spheres, however, all are found in different environments (Figure 9). The Ag1…Ag1^(iii)^, and Ag2…Ag2^(iv)^ distances are 2.8315(6) Å and 2.8310(6) Å, respectively. These distances are shorter than the Ag-Ag distance in metallic silver. The coordination environments around Ag1 and Ag2 are similar, in that they are coordinated by one carboxylate group of a phosphabetaine, one nitrogen of another phosphonium salt and one water molecule (see the respective bond distances in the legend to Figure 9). There are no interactions between Ag1–Ag4 and Ag2–Ag3 based on that their distances (3.651 Å and 3.641 Å, respectively) are close to the sum of the van der Waals radii of two Ag(I) ions (3.44 Å).

The polymer layers are divided by the coordinated triflate ions and acetone molecules that are located in the separating channels (Figures S18, S27 and S28). The resulting crystal structure consists of dense packed 2D coordination polymer layers separated by loosely filled solvent-containing voids (Figure S19). 

### 2.4. Antimicrobial Activity of the Phosphabetaines 1 and 2, and the Silver-Based Coordination Polymer CP1.2

It is known from the literature, that several Ag(I)-containing coordination polymers or networks have stronger antimicrobial effect than AgNO_3_ which is often used as a standard for antimicrobial activity [49]. Unfortunately, from the three new silver(I)-based coordination polymers, only **CP1.2** was suitable for determination of its antimicrobial effect. As a very light-sensitive compound, **CP1.1** could not be used for such studies, and we could not isolate enough quantities of **CP.2** to carry out the determination procedure. Nevertheless, we made a comparative antimicrobial study of the free ligands, PTA, **1**, and **2**, and that of **CP1.2**. The results were compared to the efficiency of AgNO_3_ and Ag(CF_3_SO_3_). 

Antimicrobial effects of the said compounds were tested on well-known, widely used and easily available representatives of three major groups of microorganisms, i.e., Gram-positive bacteria (*Bacillus subtilis*), Gram-negative bacteria (*Pseudomonas putida F1*), and unicellular fungi (*Saccharomyces cerevisiae S288C*). Each of them grows optimally in the AB medium employed, allowing comparison of the inhibitory effects. Furthermore, all three microbial strains are considered as a reference within their own biological species.

It can be seen from the data of Table 2, that PTA and the phosphabetaines **1** and **2**, have hardly any antibacterial activity. In contrast, the antibacterial activity of the coordination polymer **CP1.2** is much higher than its constituent phosphabetaine **1**. Furthermore, with *B. subtilis* and *S. cerevisiae S288C* this compound has substantially higher antimicrobial activity (expressed as MIC, nmol/mL) than AgNO_3_ or Ag(CF_3_SO_3_). Interestingly, the MIC values for an in situ mixture of Ag(CF_3_SO_3_) and **1** were basically the same than that for Ag(CF_3_SO_3_) in case of all three microorganisms. This may be the consequence of a slow in situ formation (if any) of **CP1.2** under conditions of MIC determinations. Altogether, these results support the earlier observations that silver(I)-based coordination polymers can have higher antimicrobial activities than simple silver(I) salts and therefore they are worthy of further investigations.

Antimicrobial effects of several coordination polymers formed from Ag(I)-derivatives and PTA with or without simple dicarboxylic acids (succinic, adipic, malonic, 3-phenylglutaric, etc.) have been studied already [33,34,49]. Direct comparison of the MIC values determined earlier and in this work is not possible, since the test microorganisms and also the test conditions were different. Nevertheless, our group has found CP1.2 about 2.5–4.5 times more efficient than the AgNO_3_ standard, while Kirillov and co-workers determined 2–7 times higher antimicrobial efficiency relative to AgNO_3_ in the case of the mentioned coordination polymers against *Staphylococcus aureus*, *Escherichia coli*, and *Pseudomonas aeruginosa* bacteria as well as *Candida albicans* yeast [33,34,49]. These findings allow the conclusion, that **CP1.2** shows remarkable antimicrobial activity and that the Ag(I)-based coordination polymers with phosphabetaine ligands deserve further attention as antimicrobial agents. 

## 3. Materials and Methods 

PTA was obtained according to a published synthetic procedure. [50] All other chemicals and solvents were high quality commercial products purchased from Sigma-Aldrich/Merck, St. Louis, MO, USA; VWR International, West Chester, Pennsylvania, USA; and Molar Chemicals Kft., Halásztelek, Hungary and were used without further purification. Good quality ion-exchanged water was used throughout (S ≤2 μS). Gases (Ar and N_2_) were supplied by Linde Magyarország Zrt. (Répcelak, Hungary).

### 3.1. Synthesis of the New Phosphabetaines 1 and 2

#### 3.1.1. Synthesis of 4-carboxy-3-(1,3,5-triaza-7-phosphoniatricyclo-[3.3.1.1^3,7^]dec-7-yl)butanoate (**1**)

In a 100 mL Schlenk flask, 157 mg PTA (1 mmol) and 130 mg glutaconic acid (1 mmol) were dissolved in 2.5 mL deoxygenated water. The reaction mixture was stirred magnetically for 3 h at 70 °C. The solvent was removed at reduced pressure and the off-white solid residue was washed with 10 mL chloroform, then with 3 × 10 mL acetone and finally with 2 × 10 mL diethyl ether.

Yield: 250 mg (87%).

Elementary analysis (%): Calculated for C_11_H_18_N_3_O_4_P × 0.5 H_2_O (M = 296.26): C, 44.59; H, 6.46; N, 14.18; Found (%): C, 44.45; H, 6.53; N; 14.10.

^1^H-NMR (400 MHz, D_2_O, 25 °C) δ/ppm 4.61 (*d*, ^1^*J*_PH_ = 6.2 Hz, 6H, ^+^P–C*H_2_–*N), 4.57(*d*, *J*_BA_ = 14.1 Hz, 3H, N–C*H_2_*_(ax)_–N), 4.45 (*d*, *J*_AB_ = 13.3 Hz, 3H, N–C*H_2_*_(eq)_–N), 2.80–2.92 (*m*, 1H, ^+^P–C*H*), 2.54–2.80 (*m*, 4H, ^+^P–CH–(C*H_2_*)_2_). (Figure S2)

^13^C{^1^H} NMR (90 MHz, D_2_O, 25 °C) δ/ppm 28.27 (*d*, ^1^*J*_PC_ = 34 Hz, *C*H–P^+^), 34.31 (*s,* ^–^OOC–*C*H_2_–CH–P^+^ and HOOC–*C*H_2_–*C*H–P^+^), 48.52 (*d*, ^1^*J*_PC_ = 34 Hz, ^+^P–*C*H_2_–N), 70.55 (*d*, ^3^*J*_PC_ = 9 Hz, N–*C*H_2_–N), 176.13 (*C*OOH and *C*OO^–^). (Figure S3A–C)

^31^P{^1^H} NMR (145 MHz, D_2_O, 25 °C) δ/ppm = –34.0 (*s*). (Figure S4)

MS(ESI), positive ion mode, in H_2_O, *m*/*z* [M + H]^+^ (C_11_H_19_N_3_O_4_P), Calculated: 288.1108, Found: 288.1107; [2M + H]^+^ (C_22_H_37_N_6_O_8_P_2_), Calculated: 575.2143, Found: 575.2144. (Figure S5)

Concentrated aqueous solutions of **1** were layered with 2-propanol. In 3 weeks, colorless crystals appeared which were suitable for SC-XRD measurements.

#### 3.1.2. Synthesis of 3-carboxy-2-(1,3,5-triaza-7-phosphoniatricyclo-[3.3.1.1^3,7^]dec-7-ylmethyl)-propanoate (**2**)

(a) From PTA and itaconic acid 

In a 100 mL Schlenk flask, 157 mg PTA (1 mmol) and 130 mg itaconic acid (1 mmol) were dissolved in 2.5 mL deoxygenated water. The reaction mixture was stirred magnetically for 3 h at 70 °C. The solvent was removed at reduced pressure and the off-white solid residue was dissolved in 5 mL methanol which was later removed at reduced pressure. The white product was washed with 10 mL chloroform, then with 3 × 10 mL acetone and finally with 2 × 10 mL diethyl ether. 

Yield: 201 mg (70%). 

Elementary analysis (%): Calculated for C_11_H_18_N_3_O_4_P×2H_2_O (M = 323.28): C, 40.87; H, 6.86; N, 12.99; Found: C, 40.94; H, 6.79; N; 12.85.

^1^H-NMR (400 MHz, D_2_O, 25 °C) δ/ppm 4.28–4.64 (*m*, 12H, ^+^P–C*H_2_*–N and N–C*H_2_*–N), 2.85–3.03 (*m*, 1H, ^+^P–CH_2_–C*H*), 2.17–2.82 (*m*, 4H, ^+^P–C*H_2_*–; CH–C*H_2_*–COOH). (Figure S6)

^13^C{^1^H}-NMR (90 MHz, D_2_O, 25 °C) δ/ppm 24.00 (*d*, ^1^*J*_PC_ = 39 Hz, ^+^P–*C*H_2_), 36.95 (*d*, ^3^*J*_PC_ = 5 Hz, ^+^P–CH_2_–*C*H), 37.22 (*d*, ^2^*J*_PC_ = 14 Hz, CH_2_–COO^–^), 49.14 (*d*, ^1^*J*_CP_ = 39 Hz, ^+^P–*C*H_2_–N), 70.66 (*d*, ^3^*J*_PC_ = 9 Hz, N–*C*H_2_–N), 176.36 (*s*, *C*OOH), 178.77 (*s*, *C*OO^–^). (Figure S7A–C)

^31^P{^1^H} NMR (145 MHz, D_2_O, 25 °C) δ/ppm _=_ –41.6 (s). (Figure S8)

MS(ESI), positive ion mode, in H_2_O, *m*/*z*: [M + H]^+^ (C_11_H_19_N_3_O_4_P), Calculated: 288.1108, Found: 288.1108 and [2M + H]^+^ (C_22_H_37_N_6_O_8_P_2_), Calculated: 575.2143, Found: 575.2145. (Figure S9)

Concentrated aqueous solutions of **2** were layered with 2-propanol. In 3 weeks, colorless crystals appeared which were suitable for SC-XRD measurements.

(b) From PTA and itaconic anhydride 

**2** was prepared according to the procedure in a) above, except that 112 mg (1 mmol) 2-methylenesuccinic anhydride was used instead of itaconic acid.

Yield: 206 mg (72%).

^31^P{^1^H} NMR (145 MHz, D_2_O, 25 °C) δ/ppm = –41.6 (*s*).

### 3.2. Mechanochemical Synthesis of **1**, **2**, and **3** in a Planetary Ball Mill

In separate experiments, 157 mg (1 mmol) PTA and 1-1-1 mmol glutaconic, itaconic, and maleic acid (130 mg, 130 mg, and 116 mg, respectively), were placed into a 12.5 mL RETSCH 01.462.0239, 1.4112 model stainless steel milling jar. The mixtures were milled in a ‘RETSCH PM 100′ type planetary ball mill with 6-8 pieces of stainless steel G100 ball bearings (8 mm diam.) for 4 h at 550 rpm. Cycles consisted of 2 min milling + 1 min cooling. At the end of milling, the solid product was removed from the jar by dissolving it in the minimum amount (approximately 2 mL) of water, and the white product was isolated by evaporation of the solvent. 

The products were identified by the respective ^31^P NMR spectral data which were found identical to those determined for the same phosphabetaines obtained from reactions in aqueous solutions. The spectra of isolated phosphabetaines **1**-**3** did not show presence of unreacted PTA or other P-containing impurities. Results of the syntheses:

**1**: Yield: 212 mg (74%);

^31^P NMR (145 MHz, D_2_O, 25 °C): δ /ppm = –33.95 (s). (Figure S13A)

**2**: Yield: 221 mg (77%); 

^31^P NMR (145 MHz, D_2_O, 25 °C): *δ* /ppm = –41.75 (*s*). (Figure S13B)

**3**: Yield: 248 mg (91%);

^31^P NMR (145 MHz, D_2_O, 25 °C): δ /ppm = –37.50 (s). (Figure S13C)

The same reaction was attempted with 1 mmol PTA and 1 mmol fumaric acid, however, ^1^H and ^31^P NMR spectroscopy showed mostly the resonances of the starting materials with only a 5% conversion of PTA.

### 3.3. Synthesis of Silver(I)-Based Coordination Polymer

#### 3.3.1. Synthesis of CP1.1

In a 100 mL beaker, an aqueous solution (2 mL) of 180 mg (0.7 mmol) Ag(CF_3_SO_3_) was added to 100 mg (0.35 mmol) of **1** dissolved in 5 mL water. The solution was stored in the dark at 5 °C for 2 weeks. During this time colorless, very light sensitive crystals were formed. These were isolated by filtration and stored with the exclusion of light. 

Yield (calculated for **1**): 110 mg (56%).

Elementary analysis (%): Calculated for C_12_H_20_AgF_3_N_3_O_8_PS (M = 561.05): C, 25.64; H, 3.59; N, 7.47; S, 5.70; Found: C, 23.84; H, 3.51; N, 7.29; S, 5.29.

^1^H-NMR (400 MHz, D_2_O, 25 °C) δ/ppm 4.39–4.73 (*m*, 12H, ^+^P–C*H_2_*–N and N–C*H_2_*–N), 2.78–2.90 (*m*, 1H, ^+^P–C*H*), 2.51–2.72 (*m*, 4H, ^+^P–CH–(C*H_2_*)_2_).

^31^P{^1^H} NMR (145 MHz, D_2_O): δ/ppm = –33.78 (b*s*). (Figure S10)

#### 3.3.2. Synthesis of CP1.2

In a 100 mL beaker, an aqueous solution (2 mL) of 180 mg (0.7 mmol) Ag(CF_3_ SO_3_) was added to 100 mg (0.35 mmol) of **1** dissolved in 5 mL water. The solution was layered with 2-propanol and the resulting biphase was stored in the fridge at 5 °C for 2 weeks. During this time colorless crystals were formed which were insensitive to light. These were isolated by filtration, washed with 2-propanol, and stored with the exclusion of light. 

Yield (calculated for **1**): 199 mg (87%).

Elementary analysis (%): Calculated for C_12_H_17_Ag_2_F_3_N_3_O_7_PS (651.03): C, 22.14; H, 2.63; N, 6.45; S, 4.93; Found: C, 22.43, H, 3.18, N, 6.55, S, 5.08.

^1^H-NMR (400 MHz, D_2_O, 25 °C) δ/ppm 4.41–4.73 (*m*, 12H, ^+^P–C*H_2_*–N and N–C*H_2_*–N), 2.81–2.91 (*m*, 1H, ^+^P–C*H*), 2.46–2.67 (*m*, 4H, ^+^P–CH–(C*H_2_*)_2_).

^31^P{^1^H} NMR (145 MHz, D_2_O): δ/ppm = –33.23 (*s*). (Figure S11)

#### 3.3.3. Synthesis of CP2

A concentrated aqueous solution of Ag(CF_3_SO_3_) was dropped into an aqueous solution of phosphabetaine **2** (20 mg/mL) until the reaction mixture became cloudy. The reaction mixture was filtered, the filtrate was layered with acetone and stored in the fridge. In a few days, in addition to deposition of metallic silver and a very small amount of a white powder, a few crystals of **CP2** were formed. The latter were harvested and proved to be single crystals, suitable for X-ray diffraction analysis. The crystals easily decomposed upon exposure to light and despite all attempts we could not obtain sufficient quantities of **CP2** for NMR analysis. 

### 3.4. General Methods

Elementary analyses were obtained with the use of an ElementarVario Micro (CHNS) equipment (Elementar Analysensysteme GmbH, Elementar-Straße 1, 63505 Langenselbold, Germany)

High-resolution electrospray ionization mass spectra (HR ESI-MS) were recorded on a Bruker maXis II MicroTOF-Q type Qq-TOF-MS instrument and controlled by Compass Data Analysis 4.4 software from Bruker (Bruker Daltonik, Bremen, Germany).

Reactions in a planetary ball mill were carried out with the use of a model ‘RETSCH PM 100′ (Retsch GmbH, 42781 Haan, Retsch-Alle 1-5, Germany) instrument with a stainless steel jar (12.5 mL) and G100 ball bearings (Ø 8 mm) operated at room temperature. Cycles consisted of 2 min milling + 1 min cooling time.

^1^H, ^13^C- and ^31^P{^1^H} NMR spectra were recorded on Bruker Avance DRX 360 MHz and Bruker Avance I 400 MHz spectrometers (Bruker, Billerica, MA, USA). ^1^H NMR spectra were referenced to DSS (Na-salt of 2,2-dimethyl-2-silapentane-5-sulfonate), while for the ^31^P NMR spectra 85% H_3_PO_4_ was used as reference standard. The data were analysed with the use of TOPSPIN 3.5pl5 (Academic licence) program.

Single crystal X-ray diffraction (SC-XRD) measurements were performed using a Bruker–Nonius MACH3 four-circle diffractometer equipped with a point detector, a Bruker D8 Venture diffractometer, or SuperNova X-ray diffractometer system (Rigaku Corporation, Tokyo, Japan) and the methods and software described in [51,52,53,54,55,56,57,58,59,60,61,62,63]. The crystallographic data for all compounds were deposited in the Cambridge Crystallographic Data Centre (CCDC) with the No. CCDC 2038453-203845. Details of the structure determinations are found in the Supplementary Materials. 

Antibacterial effects of the new compounds **1**, **2**, and **CP1.2** together with that of standard compounds for comparison, were determined with the use of the method of successive dilutions [64]. Cells were grown in AB culture medium (pH = 7.12) [64], at 30 °C (*Pseudomonas putida F1*, *Saccharomyces cerevisiae S288C*) and at 37 °C (*Bacillus subtilis*), respectively. Solutions of **CP1.2** were found stable in the AB culture medium as shown by the constant optical density (OD = 0.613 at λ = 430 nm, *c* = 1 mg/mL, room temperature, 16 h). In all cases, incubations lasted for 24 h. The determination of antimicrobial effect was based on the measurements of the optical density of the cell cultures at 600 nm (OD_600_). The 24 h changes in the optical density (Δ(OD_600_)/24 h) of the cultures of the three studied microorganisms in AB culture medium without any additive were 2.289 ± 0.015 (*P. putida F1*), 2.128 ± 0.055 (*B. subtilis*), and 1.898 ± 0.129 (*S. cerevisiae S288C*), respectively. MIC values are defined as the minimum concentration (μg/mL; nmol/mL; nmol Ag/mL) of the given compound which completely inhibits the growth of the bacteria or the yeast. 

The *P. putida F1* bacteria strain was kindly provided by Prof. David Gibson, University of Iowa, IA, USA) to Prof. Levente Karaffa’s laboratory (Department of Biochemical Engineering, University of Debrecen, Hungary) and was maintained and grown in BSM culture medium. [65] The *B. subtilis* strain was also provided by Prof. Karaffa’s laboratory, while the *S. cerevisiae S288C* strain [66] was made available for the studies by the Department of Genetics and Applied Microbiology, University of Debrecen, Hungary). 

## 4. Conclusions

The reaction of unsaturated 5-carbon aliphatic diacids, *trans*-2-glutaconic and itaconic acid with 1,3,5-triaza-7-phosphaadamantane (PTA) yield zwitterionic P-alkylated compounds (phosphabetaines **1** and **2**, respectively). In addition to their synthesis in solution, **1** and **2**, as well as the analogous phosphabetaine **3** (obtained from PTA and maleic acid), the mechanochemical synthesis in a planetary ball mill also proved to be highly efficient and led to isolated yields of 74–91%. These compounds contain two carboxylate groups and three nitrogen atoms in the same molecule which make them excellent candidates for building up coordination polymers. In the reaction of Ag(CF_3_SO_3_) and **1**, two such coordination polymers—**CP1.1** and **CP1.2**—were obtained, while the reaction with **2** led to formation of **CP2**. **CP1.1** and **CP1.2** were thoroughly characterized in solution, and the solid state structures of all three coordination polymers were determined with single-crystal X-ray diffraction. The structural studies revealed interesting features of the 3D polymer structures, such as Ag-Ag- interactions and solvate channels. In addition, an investigation into the antibacterial effects of **1**, **2**, and **CP1.2** showed that the coordination polymer **CP1.2** had 2–4 times higher antibacterial activity against the Gram-positive bacterium, *Bacillus subtilis*, as well as against the yeast species *Saccharomyces cerevisiae S288C* than Ag(CF_3_SO_3_) or AgNO_3_. AgNO_3_ is often used as standard in determination of minimum inhibitory concentration of Ag-based antibacterial agents. Based on these results, additional studies on the coordination chemistry, solid state structures and biological activity of the phosphabetaines obtained from PTA and their metal complexes may lead to further interesting and remarkable discoveries in inorganic and materials chemistry and in antimicrobial applications.

## Figures and Tables

**Table 1:** Yields of the resulting phospabetaines in the reaction of PTA with unsaturated dicarboxylic acids in aqueous solution and in planetary ball mill

	Solution Synthesis		Mechanochemical Synthesis	
Dicarboxylic acid (# of phosphabetaine)	Conversion,% (reaction time, h)	Yield (%) [Reference]	Conversion,% (milling time, h)	Yield (%)
Glutaconic (**1**)	100 (3)	87 ^a^ [this work]	100 (4)	74
Itaconic (**2**)	100 (2)	71 ^a^ [this work]	100 (4)	77
Maleic (**3**)	100 (3)	74 ^b^ [38]	100(4)	91
Citraconic (**4**)	n.d.	40 ^c^ [38]	5 (8)	n.d.

Reaction conditions: Aqueous synthesis: 1 mmol PTA, 1 mmol unsaturated dicarboxylic acid, 2.5 mL water; ^a^ 70 °C, 3 h; ^b^ 1 °C, 3 h; ^c^ 70 °C, 48 h; Mechanochemical synthesis: 1 mmol PTA, 1 mmol unsaturated dicarboxylic acid; 12.5 mL milling jar, 8 stainless steel milling balls Ø 8 mm, 550 rpm, 4 h, 80 cycles (2 min milling + 1 min cooling). Conversions determined from ^1^H NMR spectra of the reaction mixtures.

**Table 2:** Antimicrobial activity of PTA, phosphabetaines **1** and **2**, and the coordination polymer **CP1.2** expressed in minimum inhibition concentrations (MIC)

Antimicrobial Agent	MIC ^a^		
	*Pseudomonas putida F1*	*Bacillus subtilis*	*Saccharomyces* *cerevisiae S288C*
PTA	723	647	286
	114 ^b^	102 ^b^	45 ^b^
**1**	422	264	385
	122 ^b^	76 ^b^	111 ^b^
**2**	443	269	348
	128 ^b^	77 ^b^	100 ^b^
**CP1.2**	86	23	55
	56 ^b^	15 ^b^	36 ^b^
Ag(CF_3_SO_3_) + **1**	77	100	135
	51 ^b^	66 ^b^	89 ^b^
AgNO_3_	77	100	135
	13 ^b^	17 ^b^	23 ^b^
Ag(CF_3_SO_3_)	77	100	143
	20 ^b^	26 ^b^	37 ^b^

*Conditions:* AB culture medium; T = 30 °C, *P. putida F1*, *S. cerevisiae S288C*; T = 37 °C, *Bacillus subtilis*. ^a^ nmol/mL, ^b^ μg/mL.

## Supplementary Materials

The following are available online. Time-dependent ^1^H NMR spectra of a mixture of PTA and itaconic acid; ^1^H, ^13^C, and ^31^P NMR spectra of **1**, **2**, **3**, **CP1.1**, **CP1.2**; MS(ESI) spectra of **1**, **2**; Experimental data for SC-XRD structure determinations; Crystal data and details of measurements for **1**, **2**, **CP1.1**, **CP1.2**, **CP.2**; Selected bond lengths and angles of PTA and its derivatives; ORTEP diagrams of **1**, **2**, **CP1.1**, **CP1.2**, **CP.2**; Selected hydrogen bond lengths and packing diagrams for **1**, **2**, **CP1.1**, **CP1.2**; Views of solvent molecules, anions and voids in the crystal lattices of **2**, **CP1.1**, **CP1.2**, **CP2**.
