# Supplementary material for: Mechanochemical P-derivatization of 1,3,5-Triaza-7-Phosphaadamantane (PTA) and Silver-Based Coordination Polymers Obtained from the Resulting Phosphabetaines
Source: Molecules. 2020 Nov 16;25(22):5352. doi: 10.3390/molecules25225352 (PMC7697749; doi:10.3390/molecules25225352)
Supplement: Supplementary file 1 [file molecules-25-05352-s001.pdf]

Supplementary Materials

to

Mechanochemical P-derivatization of 1,3,5-Triaza-7-Phosphaadamantane (PTA) and Silver-Based Coordination Polymers Obtained from the Resulting Phosphabetaines

Antal Udvardy [1]*, Csenge Tamara Szolnoki [1,2], Réka Gombos [1], Gábor Papp [1], Éva Kováts [3], Ferenc Joó [1,4]*, Ágnes Kathó [1]

[1] Department of Physical Chemistry, University of Debrecen, P.O. Box 400, H-4002 Debrecen, Hungary; szolnoki.csenge@science.unideb.hu (Cs.T. Sz.), gombos.reka@science.unideb.hu (R.G.), papp.gabor@science.unideb.hu (G.P.), katho.agnes@science.unideb.hu (Á.K.)

[2] Doctoral School of Chemistry, University of Debrecen, P.O. Box 400, H-4002 Debrecen, Hungary

[3] Institute for Solid State Physics and Optics, Wigner Research Centre for Physics, Konkoly Thege Miklós u. 29-33, H-1121 Budapest, Hungary

[4] MTA-DE Redox and Homogeneous Catalytic Reaction Mechanisms Research Group, P.O. Box 400, H-4002 Debrecen, Hungary (kovats.eva@wigner.mta.hu)

*****Correspondence: udvardya@unideb.hu (A. U.); joo.ferenc@science.unideb.hu (F. J.)

Table of Contents

**Figure S1.** Time-dependent ^{1}H-NMR spectra of a mixture of PTA and itaconic acid	p3
**Figures S2-S4.** ^{1}H, ^{13}C, and ^{31}P-NMR spectra of **1.**	p4-8
**Figure S5.** MS(ESI) spectrum of **1.**	p9
**Figures S6-S8.** ^{1}H, ^{13}C, and ^{31}P-NMR spectra of **2.**	p10-14
**Figure S9.** MS(ESI) spectrum of **2.**	p15
**Figures S10-S11.** ^{31}P-NMR spectra of **CP1.1, CP1.2**	p16
**Figure S12**. Overlaid ^{1}H-NMR spectra of aqueous solutions of **1, CP1.1, C1.2.**	p17
**Figures S13A-S13C.** ^{31}P-NMR spectra of **1-3**, synthenized by a planetary ball mill	p18-19
Experimental data for SC-XRD structure determinations	p20
**Table S1.** Crystal data and details of measurements for **1**, **2**, **CP1.1**, **CP1.2**, **CP.2**	p21
**Table S2**. Selected bond lengths and angles of PTA and its derivatives	p23
**Figure S14**. ORTEP diagram of the asymmetric unit of **1**$\times H_2O$	p24
**Table S3**. Selected hydrogen bonds (including weak C–H…O interactions) in **1**	p24
**Figures S15-S16.** Partial packing views of **1.**	p25
**Figure S17.** ORTEP diagram of the asymmetric unit of **2**$\times 2\ H_2O$	p26
**Table S4.** Hydrogen bonds (including weak C–H…O interactions) in **2**	p26
**Figure S18.** Packing diagrams of **2** along the axes „**a**”, „**b**”, and „**c**”.	p27
**Figure S19.** Water molecules in **2** along axis „**c**”.	p28
**Figure S20.** ORTEP diagram of the asymmetric unit of **CP1.1**	p29
**Figure S21.** Partial packing view of **CP1.1**	p29
**Table S5.** Hydrogen bonds (including weak C–H…O interactions) in **CP1.1**	p30
**Figure S22**. Packing diagrams of **CP1.1**	p30
**Figure S23.** Triflate anions in **CP1.1** along axes „**a**” and **„c”**	p31
**Figure S24.** ORTEP diagram of the asymmetric unit of **CP1.2**	p32
**Table S6.** Hydrogen bonds (including weak C–H…O interactions) in **CP1.2**	p32
**Figure S25.** Voids in **CP1.2**	p33
**Figure S26.** ORTEP diagram of the asymmetric unit of **CP2**	p34
**Figure S27** Partial view of the crystal lattice of **CP2** showing the channels of acetone	p34
**Figure S28.** Triflate anions in **CP2** along axis „**a**”	p35

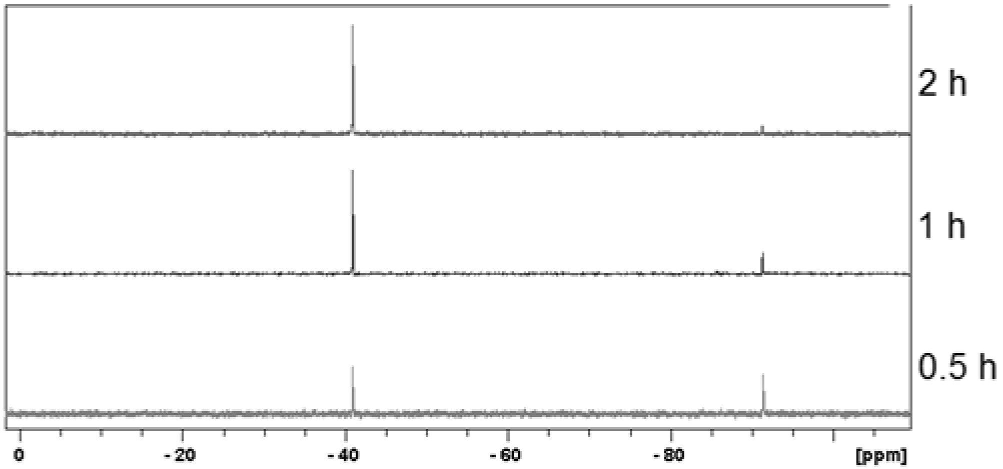

Figure S1. ^{31}P-NMR spectra of the aqueous reaction mixtures containing equivalent amounts of PTA and itaconic acid as a function of time. *Condition*s: PTA (157 mg, 1.0 mmol) and itaconic acid (130 mg, 1.0 mmol) in 2.5 mL water, T = 70 °C

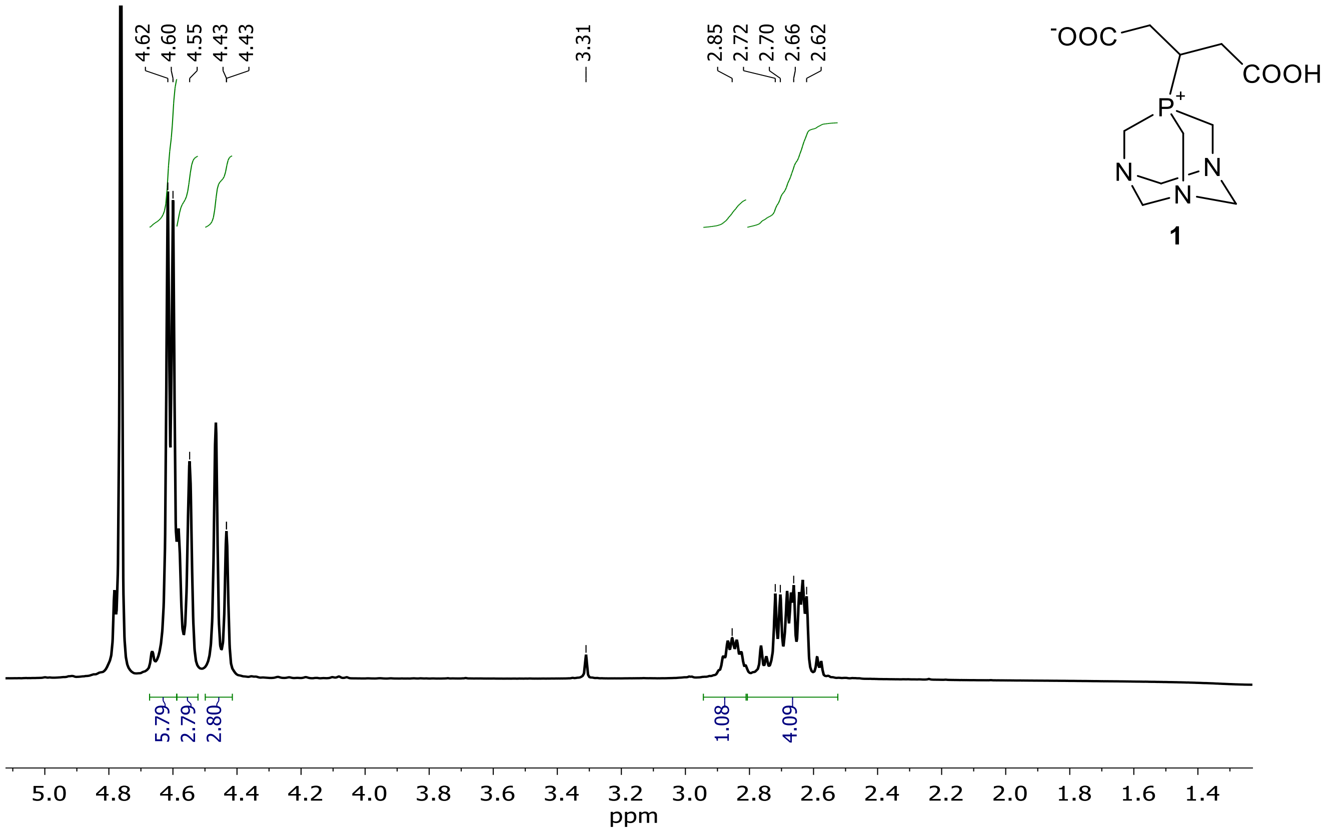

Figure S2. ^{1}H-NMR spectrum of **1**.

^{1}H NMR (400 MHz, D_2O, 25 °C): δ 4.61 (*d*, $^1J_{PH}$=6.2 Hz, 6H, $^+$P–CH_2–N), 4.57 (*d*, J_{BA}=14.1 Hz, 3H, N–C$H_{2(ax)}$–N), 4.45 (*d*, J_{AB}= 13.3 Hz, 3H, N–C$H_{2(eq)}$–N), 2.80–2.92 (*m*, 1H, $^+$P–C*H*), 2.54–2.80 (*m*, 4H, $^+$P–CH–(CH_2)$_2$) ppm.

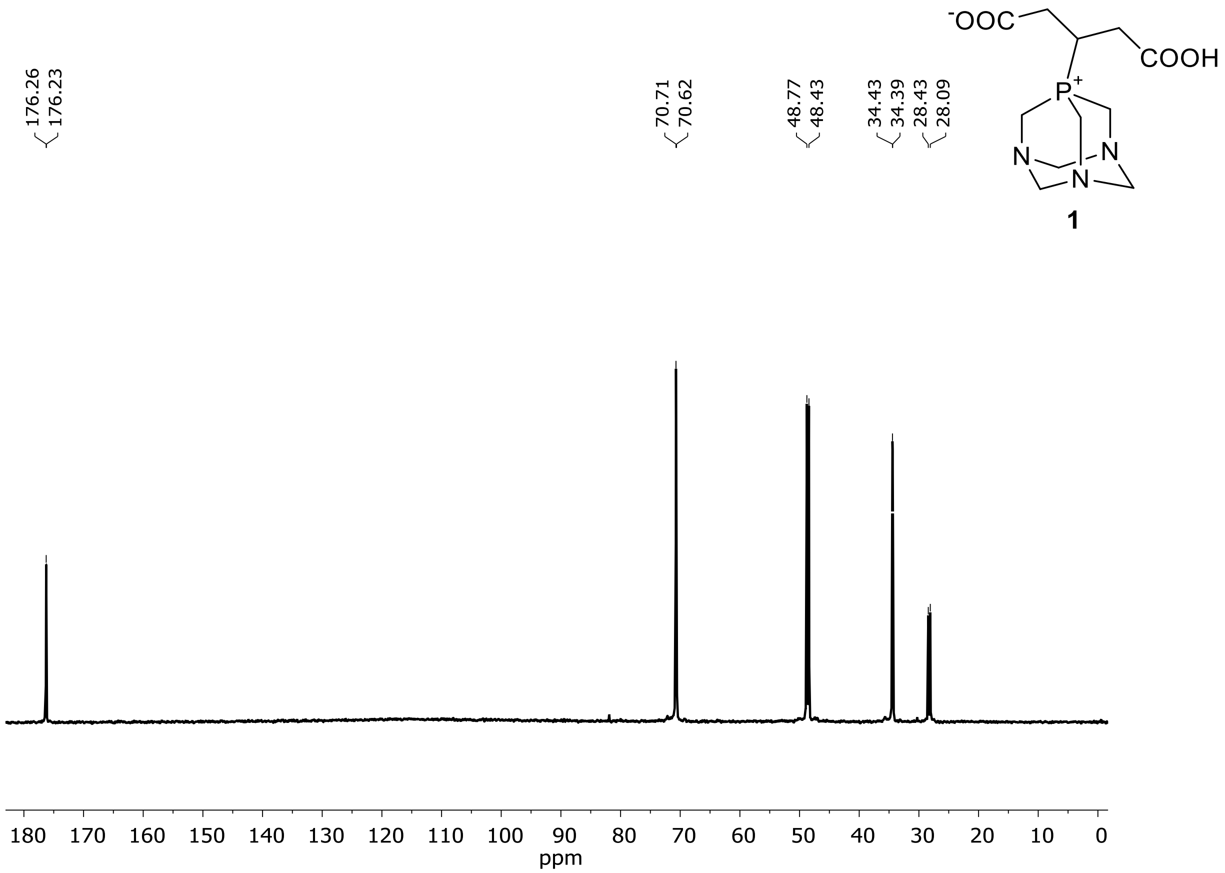

Figure S3A. $^{13}C\{^{1}H\}$-NMR spectrum of **1**.

$^{13}C\{^{1}H\}$-NMR (90 MHz, D_2O, 25 °C): δ 28.26 (*d*, $^{1}J_{PC}$ = 34 Hz, *C*H–P^+), 34.41 (*d*, $^{2}J_{PC}$ = 3 Hz, $^{-}$OOC–*C*H_2–CH–P^+ and HOOC–*C*H_2–CH–P^+), 48.60 (*d*, $^{1}J_{PC}$ = 34 Hz, $^{+}$P–*C*H_2–N), 70.67 (*d*, $^{3}J_{PC}$ = 9 Hz, N–*C*H_2–N), 176.25 (*d*, $^{3}J_{PC}$ = 3 Hz, *C*OOH, *C*OO^-) ppm.

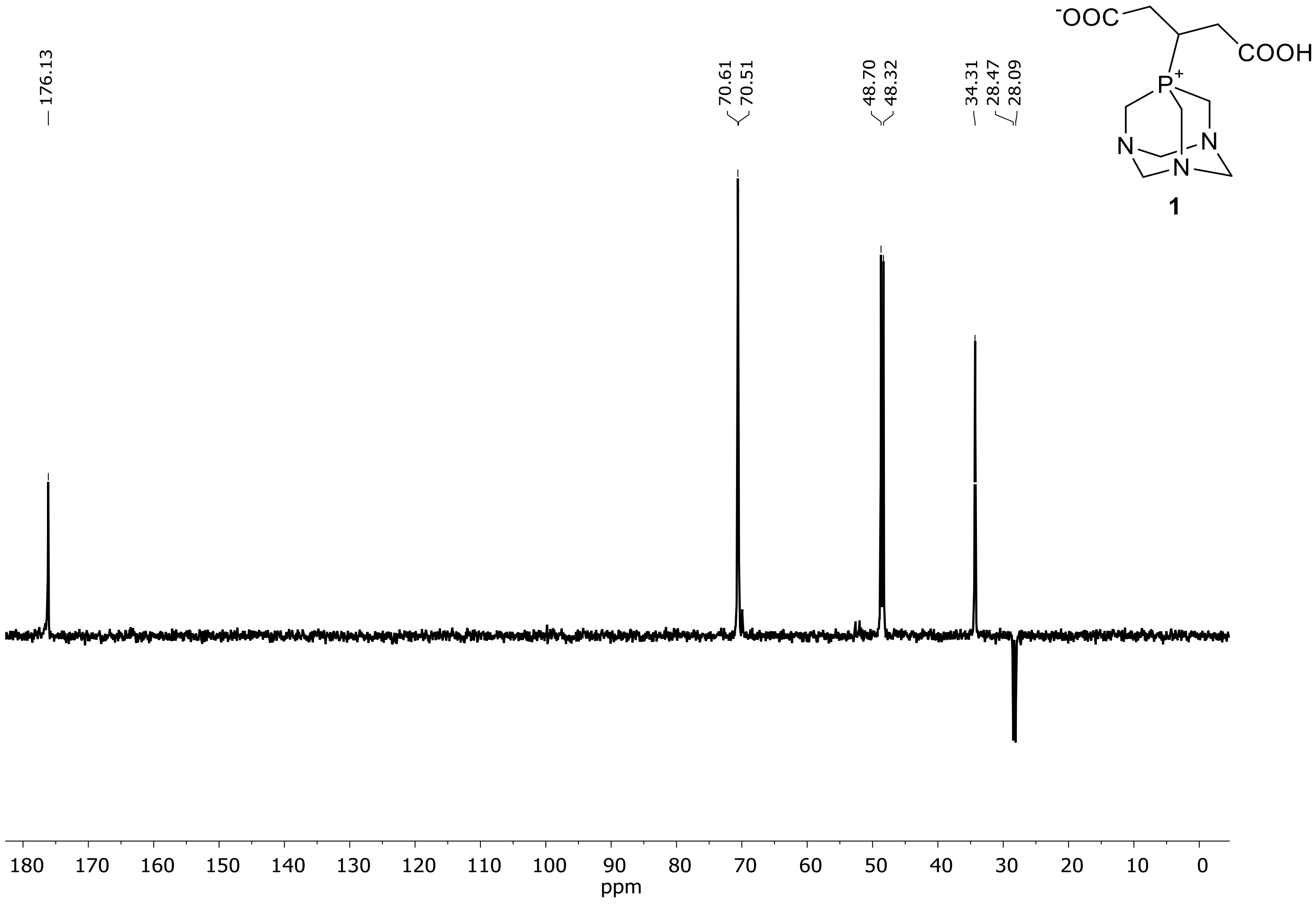

Figure S3B. $^{13}C\{^{1}H\}$-NMR spectrum of **1**.

$^{13}C\{^{1}H\}$-NMR (90 MHz, D_2O, 25 °C): δ 28.27 (*d*, $^{1}J_{PC}$ = 34 Hz, *C*H–P^+), 34.31 (*s*, $^{-}$OOC–*C*H_2–CH–P^+ and HOOC–*C*H_2–CH–P^+), 48.52 (*d*, $^{1}J_{PC}$ = 34 Hz, $^{+}$P–*C*H_2–N), 70.55 (*d*, $^{3}J_{PC}$ = 9 Hz, N–*C*H_2–N), 176.13 (*C*OOH, *C*OO^-) ppm.

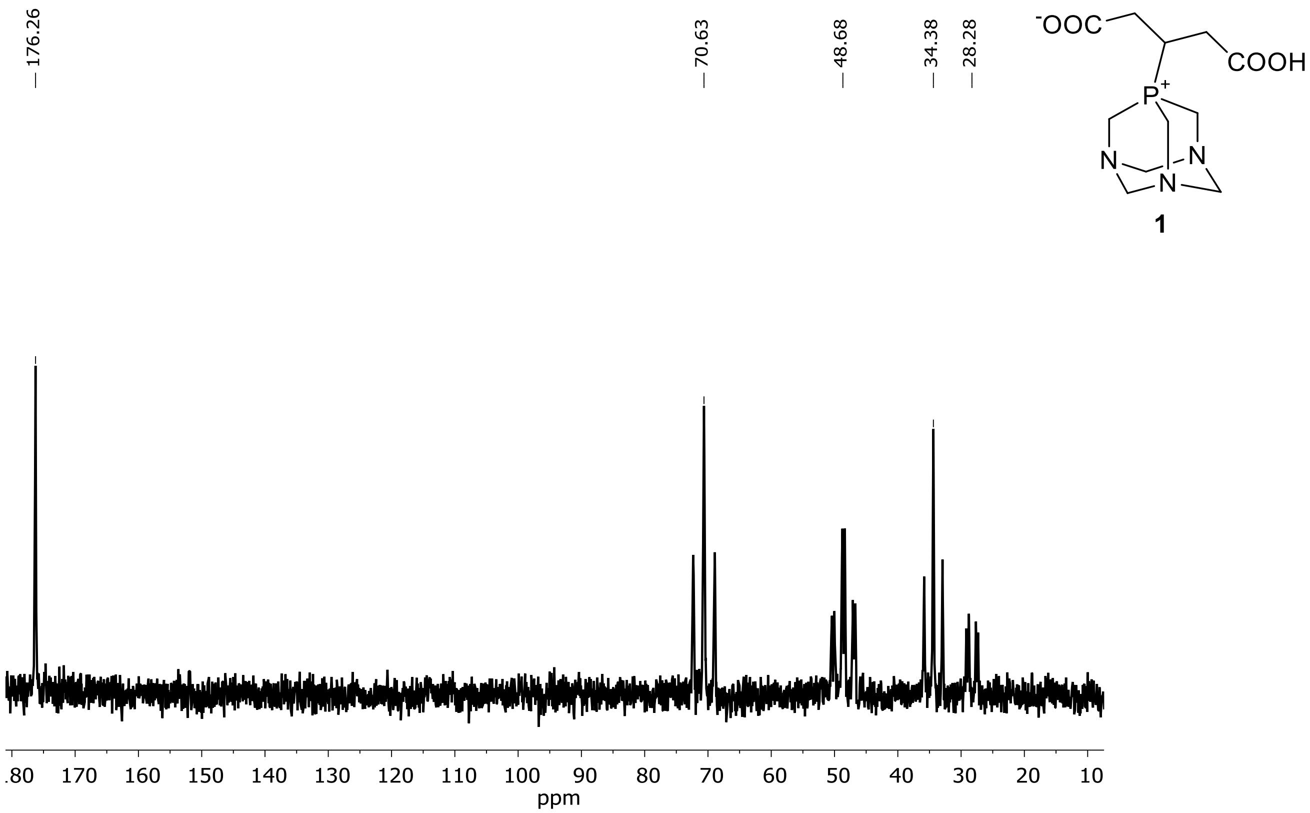

Figure S3C. ^{13}C-NMR spectrum of **1.**

^{13}C NMR (90 MHz, D_2O, 25 °C): δ 28.26 (*dd*, $^{1}J_{CH}$ = 140 Hz; $^{1}J_{PC}$ = 33 Hz $^{+}$P–*C*H), 34.38 (*t*, $^{1}J_{CH}$ = 134 Hz, $^{-}$OOC–*C*H_2–CH_2–P^+ and HOOC–*C*H_2–CH–P^+), 48.63 (*td*, $^{1}J_{CH}$ = 151 Hz; $^{1}J_{CP}$ = 35 Hz, P^+–*C*H_2–N), 70.63 (*t*, $^{1}J_{CH}$ = 150 Hz; $^{3}J_{CP}$ = 9 Hz, N–*C*H_2–N), 176.25 (*s*, *C*OOH, *C*OO^-) ppm.

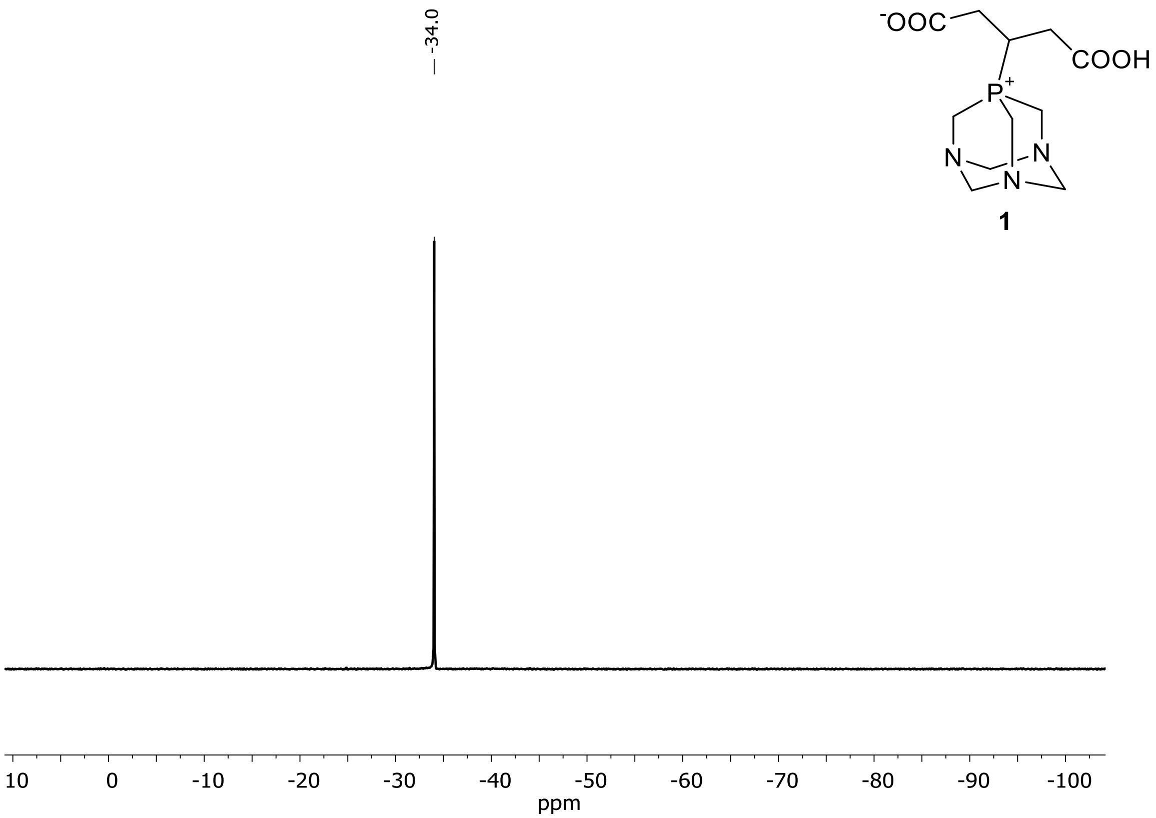

1

Figure S4. ^{31}P-NMR spectrum of **1**.

^{31}P{^{1}H}-NMR (145 MHz, D_2O, 25 °C): δ –34.0 (*s*) ppm.

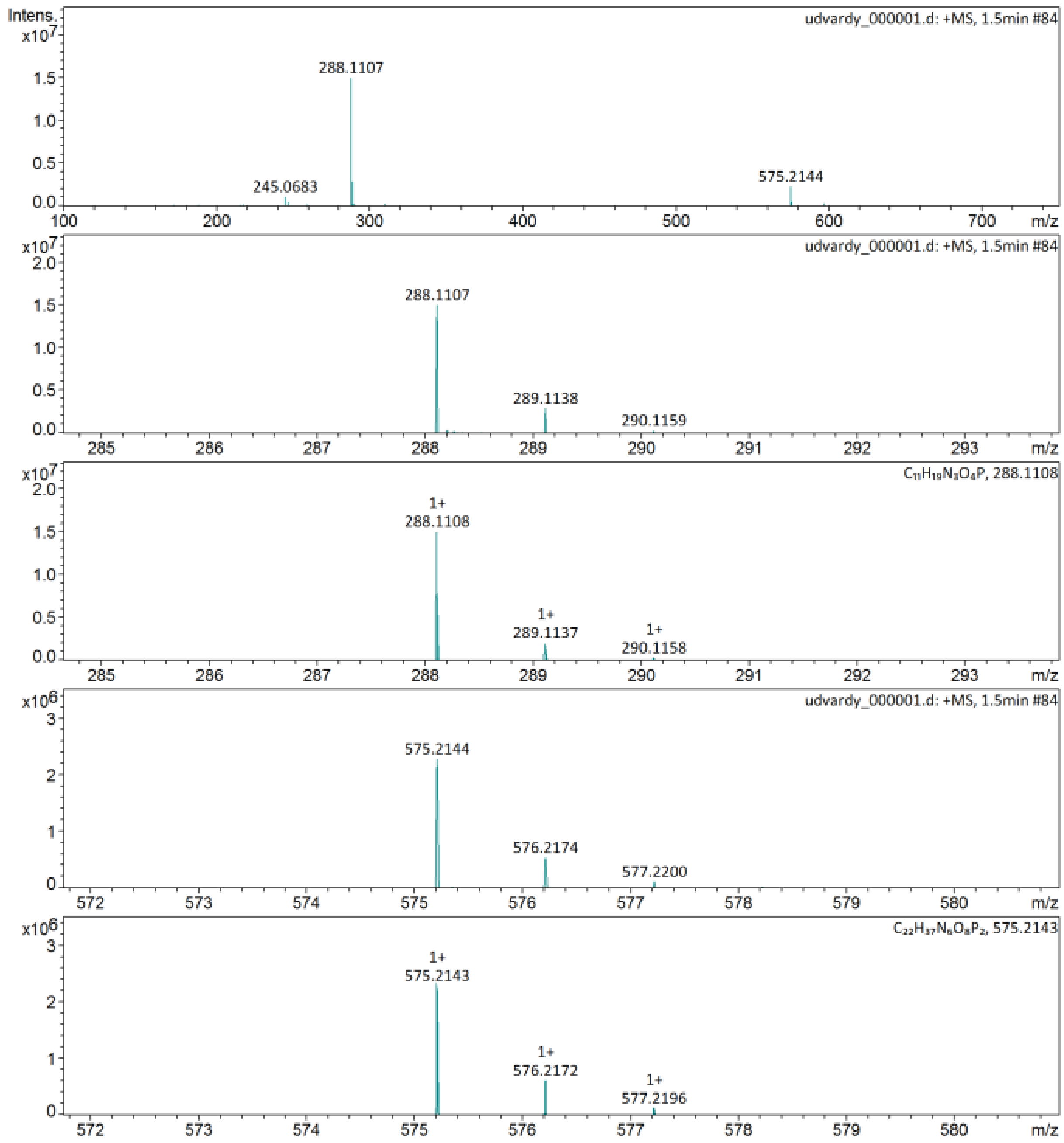

Figure S5. MS(ESI), positive ion mode, in H_2O, *m/z* for (**1**) $[M+H]^+$ ($C_{11}H_{19}N_3O_4P$), Calculated: 288.1108, Found: 288.1107 and $[2M+H]^+$ ($C_{22}H_{37}N_6O_8P_2$), Calculated: 575.2143, Found: 575.2144.

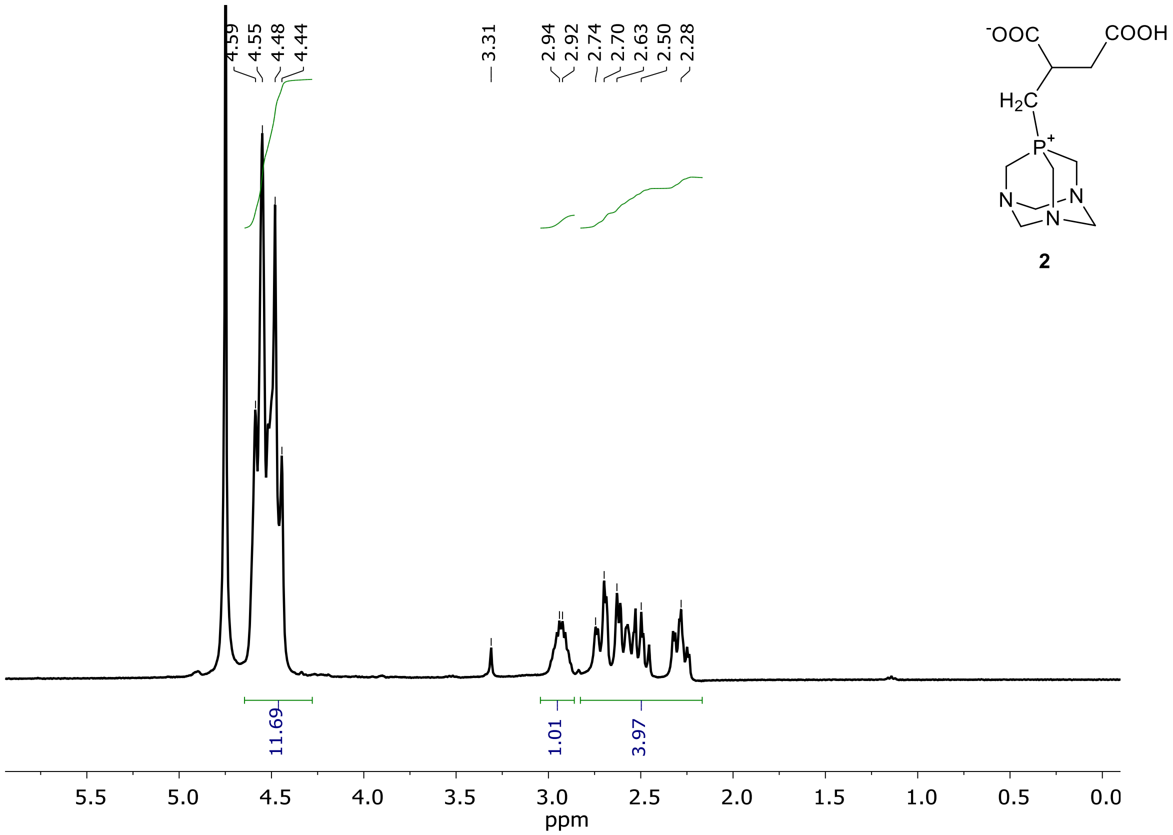

Figure S6. ^{1}H-NMR spectrum of **2.**

^{1}H-NMR (400 MHz, D_2O, 25 °C): δ 4.28–4.64 (*m*, 12H, $^{+}$P–C*H₂*–N and N–C*H₂*–N), 2.85–3.03 (*m*, 1H, $^{+}$P–CH_2–C*H*), 2.17–2.82 (*m*, 4H, $^{+}$P–C*H₂*–; CH–C*H₂*–COOH) ppm.

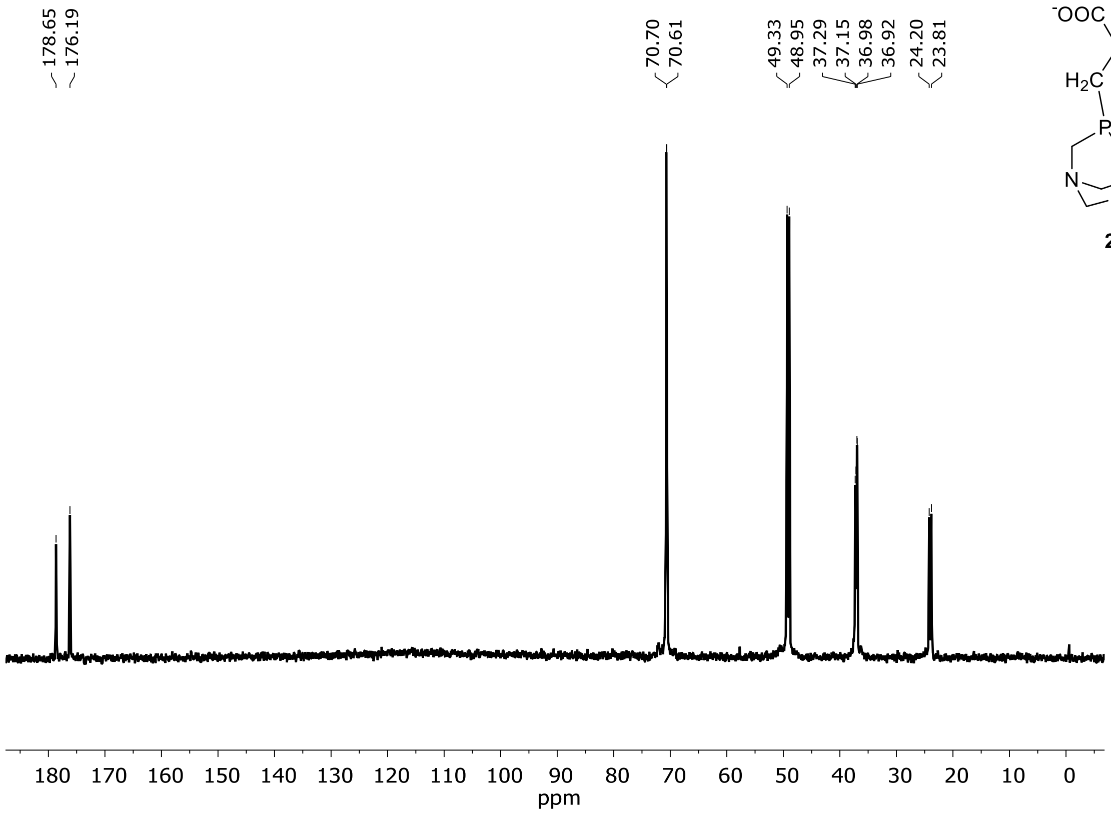

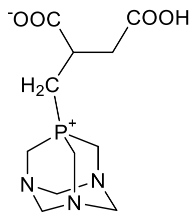

2

Figure S7A. $^{13}C\{^1H\}$-NMR spectrum of **2**.

$^{13}C\{^1H\}$ NMR (90 MHz, D_2O, 25 °C): δ 24.00 (*d*, $^1J_{PC}$= 39 Hz, $^+$P–*C*H_2–), 36.95 (*d*, $^3J_{PC}$= 5 Hz, $^+$P–CH_2–*C*H), 37.12 (*d*, $^2J_{PC}$= 14 Hz, *C*H_2–COO^-), 49.14 (*d*, $^1J_{CP}$= 39 Hz, $^+$P–*C*H_2–N), 70.66 (*d*, $^3J_{PC}$= 9 Hz, N–*C*H_2–N), 176.19 (b*d*, *C*OOH), 178.65 (*s*, *C*OO^-) ppm.

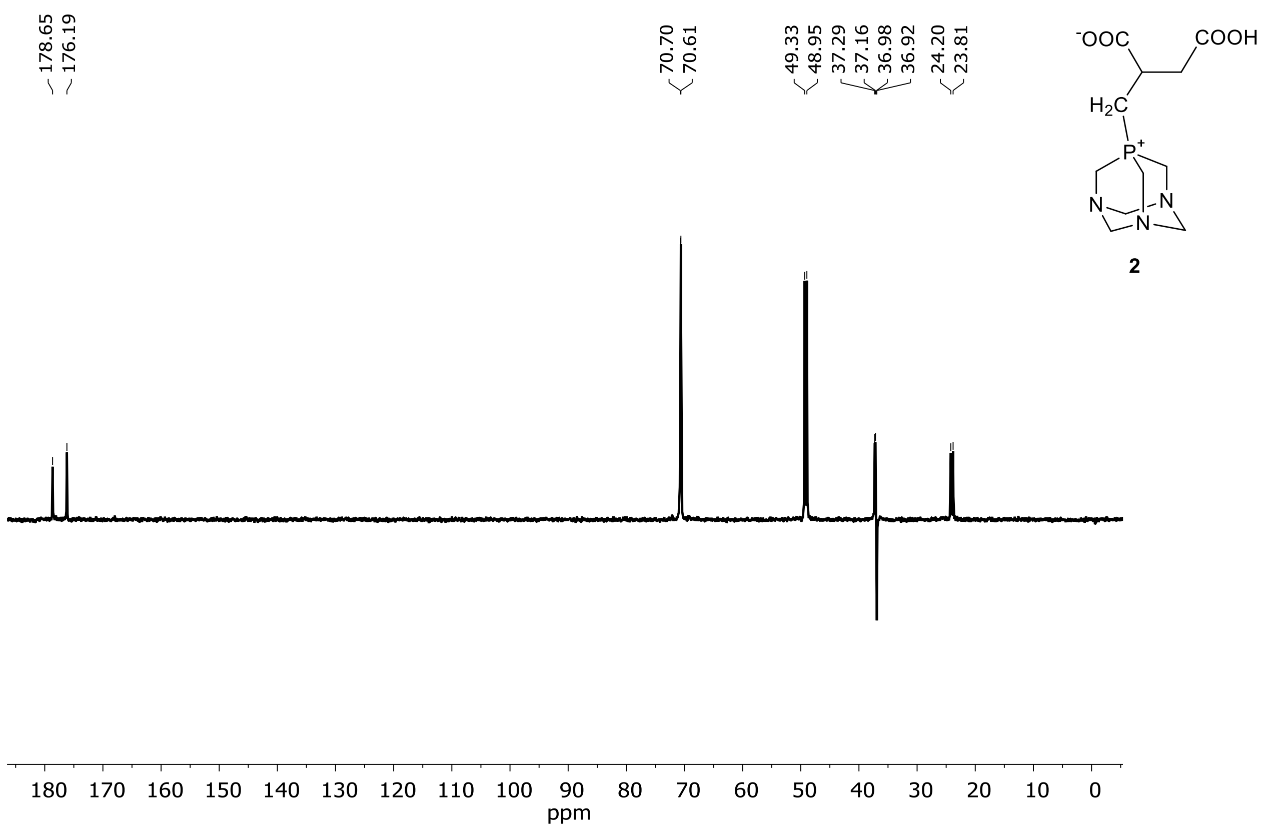

Figure S7B. $^{13}C\{^{1}H\}$-NMR spectrum of **2**.

$^{13}C\{^{1}H\}$-NMR (90 MHz, D_2O, 25 °C): δ 24.00 (*d*, $^{1}J_{PC}$= 39 Hz, $^{+}P–CH_2$), 36.95 (*d*, $^{3}J_{PC}$= 5 Hz, $^{+}P–CH_2–CH$), 37.22 (*d*, $^{2}J_{PC}$= 14 Hz, $CH_2–COO^{–}$), 49.14(*d*, $^{1}J_{CP}$= 39 Hz, $^{+}P–CH_2–N$), 70.66 (*d*, $^{3}J_{PC}$= 9 Hz, $N–CH_2–N$), 176.36 (*s*, *C*OOH), 178.77 (*s*, $COO^{–}$) ppm.

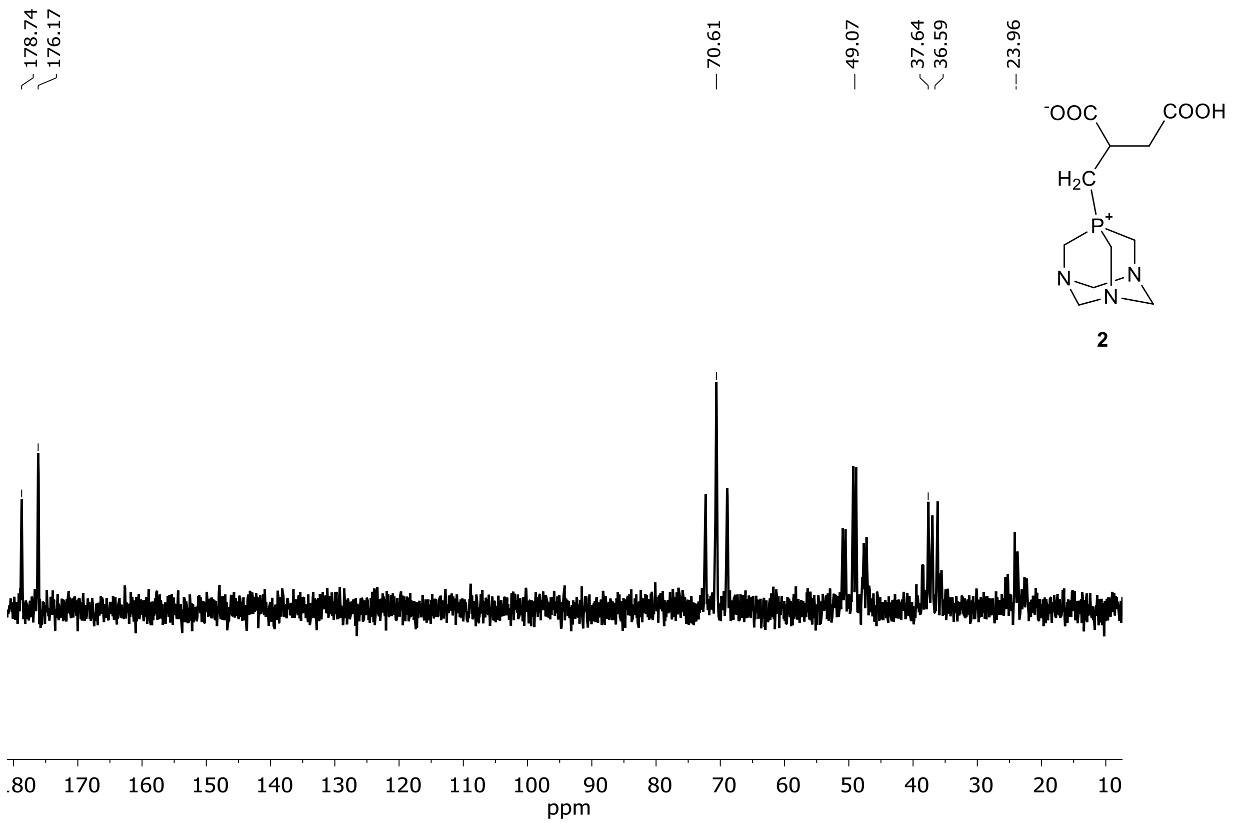

Figure S7C. ^{13}C-NMR spectrum of **2**.

^{13}C–NMR (90 MHz, D_2O, 25 °C): δ 23.96 (*td*, $^1J_{CH}$ = 137 Hz; $^1J_{CP}$ = 38 Hz, $^+$P–*C*H_2), 36.95 (*td*, $^1J_{CH}$ = 137 Hz; $^3J_{CP}$ = 6 Hz, $^+$P–CH_2–*C*H), 37.12 (d*d*, $^1J_{CH}$ = 135 Hz; $^2J_{CP}$ = 15 Hz, *C*H_2–COO^-), 49.07 (*td*, $^1J_{CH}$ = 138 Hz; $^1J_{CP}$ = 38 Hz, $^+$P–*C*H_2–N), 70.61 (*t*, $^1J_{CH}$ = 154 Hz; $^3J_{CP}$ = 9 Hz, N–*C*H_2–N), 176.19 (*s*, *C*OOH), 178.65 (*s*, *C*OO^-) ppm.

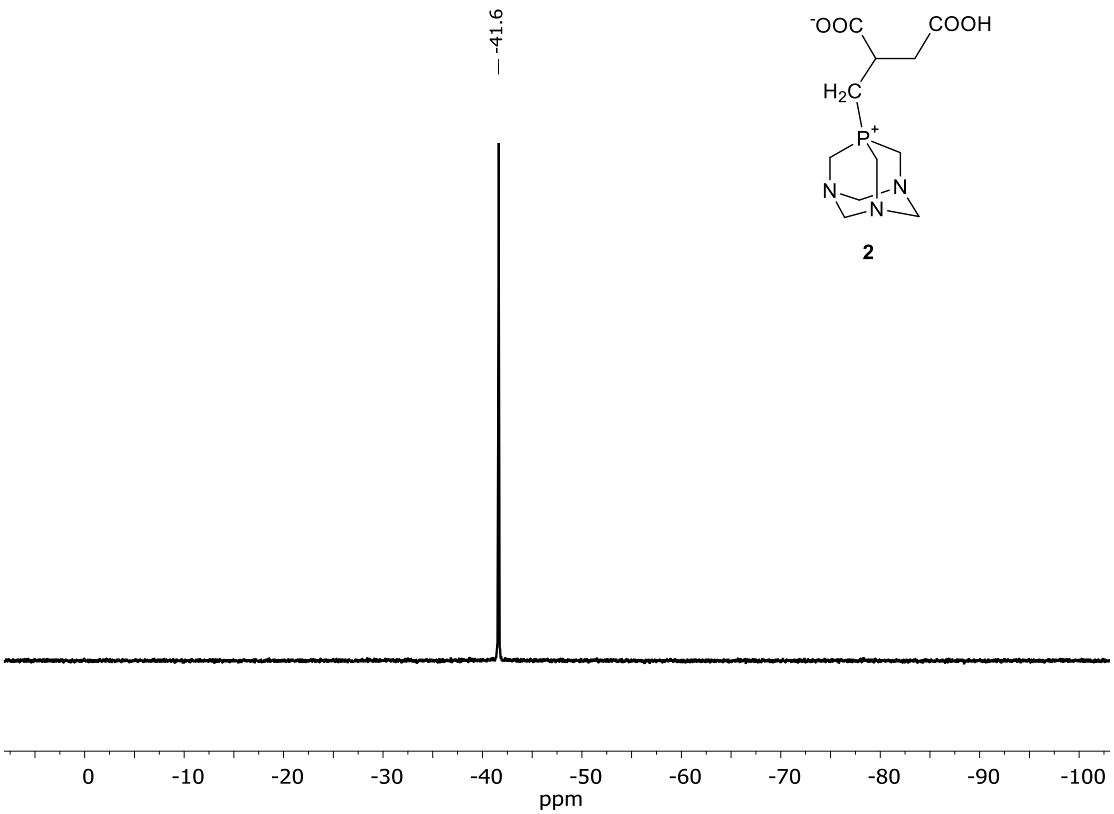

Figure S8. ^{31}P-NMR spectrum of **2**.

^{31}P{^{1}H}-NMR (145 MHz, D_2O, 25 °C): δ –41.6 (*s*) ppm.

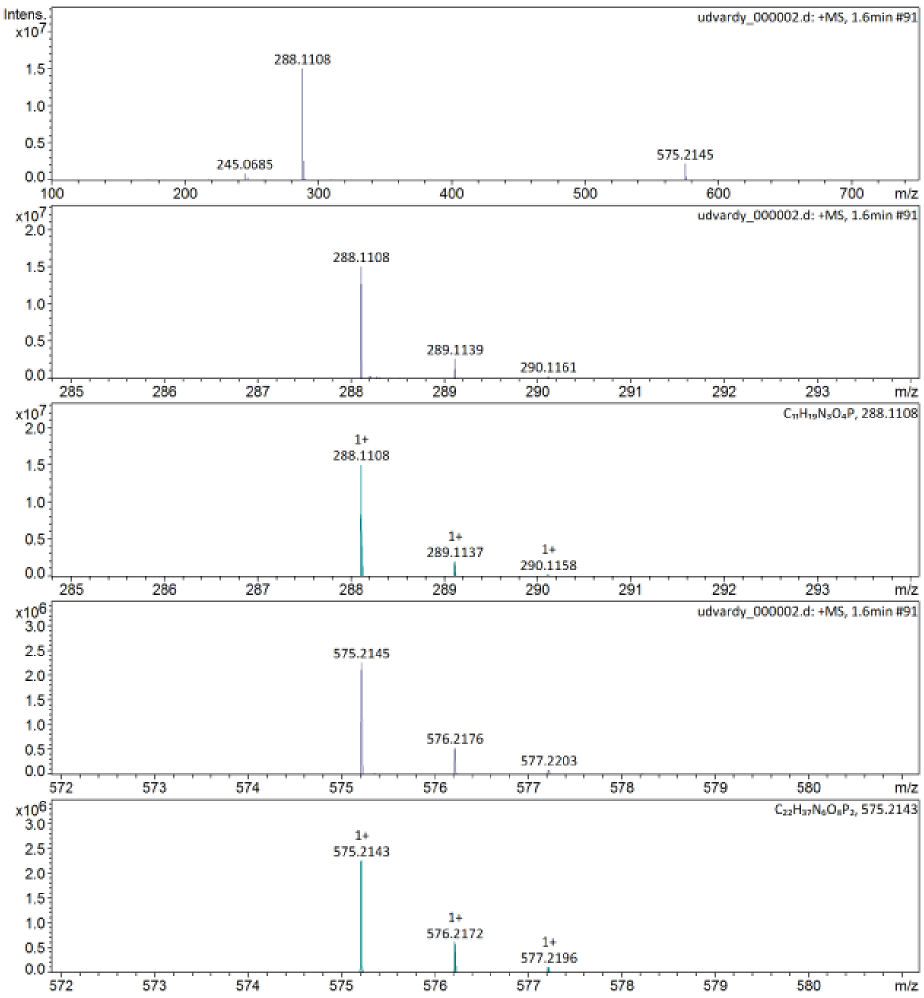

Figure S9. MS(ESI), positive ion mode, in H_2O, *m/z* for (**2**) $[M+H]^+$ ($C_{11}H_{19}N_3O_4P$), Calculated: 288.1108, Found: 288.1108 and $[2M+H]^+$ ($C_{22}H_{37}N_6O_8P_2$), Calculated: 575.2143, Found: 575.2145.

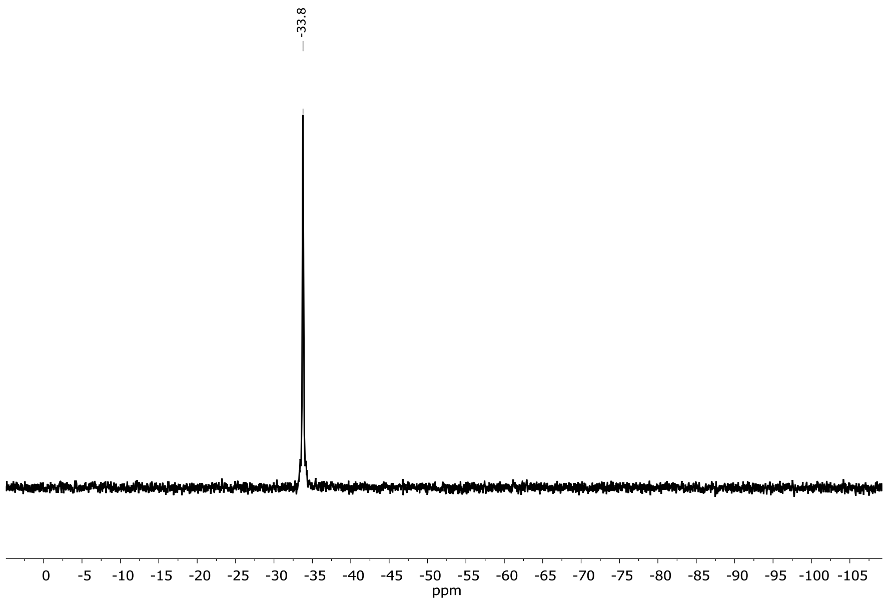

Figure S10. ^{31}P-NMR spectrum of aqueous solution of **CP1.1.**

^{31}P{^{1}H}-NMR (145 MHz, D_2O, 25 °C): δ –33.78 (*s*) ppm.

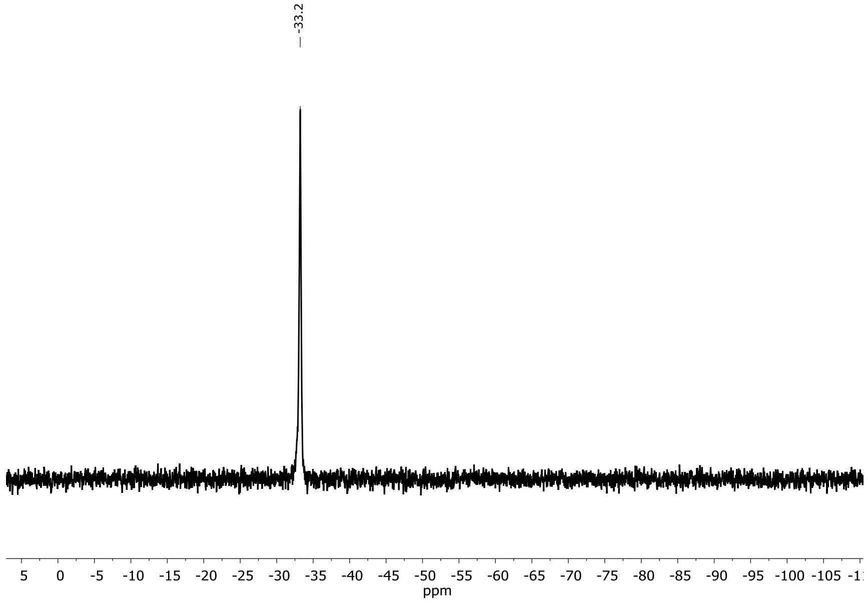

Figure S11. ^{31}P-NMR spectrum of aqueous solution of **CP1.2.**

^{31}P{^{1}H}-NMR (145 MHz, D_2O, 25 °C): δ –33.23 (*s*) ppm.

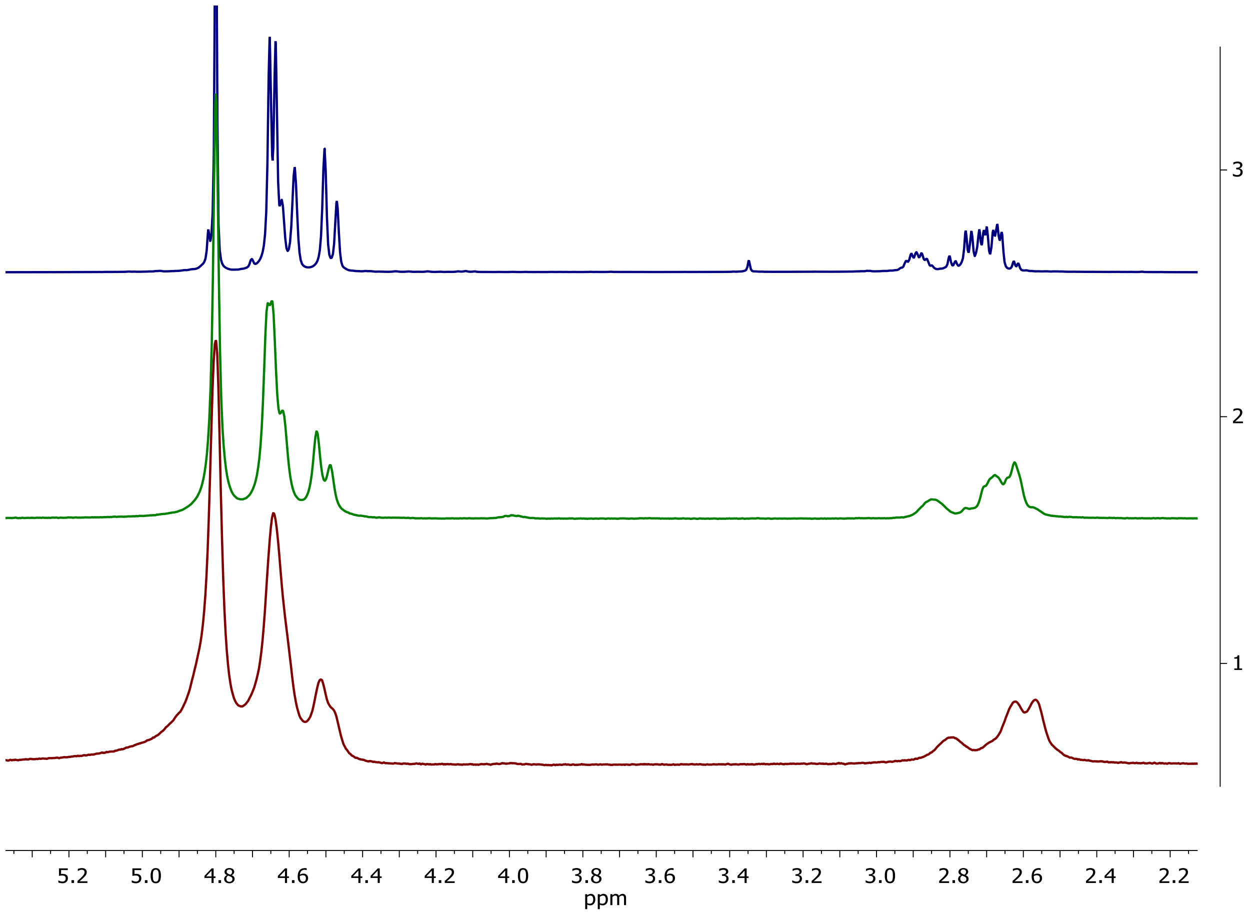

Figure S12. Overlaid ^{1}H-NMR spectra of aqueous solutions of **1, CP1.1, C1.2.**

3: ^{1}H-NMR (400 MHz, D_2O, 25 °C): δ 4.61 (*d*, $^{1}J_{PH}$=6.2 Hz, 6H, $^{+}$P–CH_2–N), 4.57(*d*, J_{BA}=14.1 Hz, 3H, N–C$H_{2(ax)}$–N), 4.45 (*d*, J_{AB}= 13.3 Hz, 3H, N–C$H_{2(eq)}$–N), 2.80–2.92 (*m*, 1H, $^{+}$P–C*H*), 2.54–2.80 (*m*, 4H, $^{+}$P–CH–(CH_2)$_2$) ppm. (**1**)

2: ^{1}H-NMR (400 MHz, D_2O, 25 °C): δ 4.41–4.73 (*m*, 12H, $^{+}$P–CH_2–N and N–CH_2–N), 2.81–2.91 (*m*, 1H, $^{+}$P–C*H*), 2.46–2.67 (*m*, 4H, $^{+}$P–CH–(CH_2)$_2$) ppm. (CP**1.2**)

1: ^{1}H-NMR (400 MHz, D_2O, 25 °C): δ 4.39–4.73 (*m*, 12H, $^{+}$P–CH_2–N and N–CH_2–N), 2.78–2.90 (*m*, 1H, $^{+}$P–C*H*), 2.51–2.72 (*m*, 4H, $^{+}$P–CH–(CH_2)$_2$) ppm. (CP**1.1**)

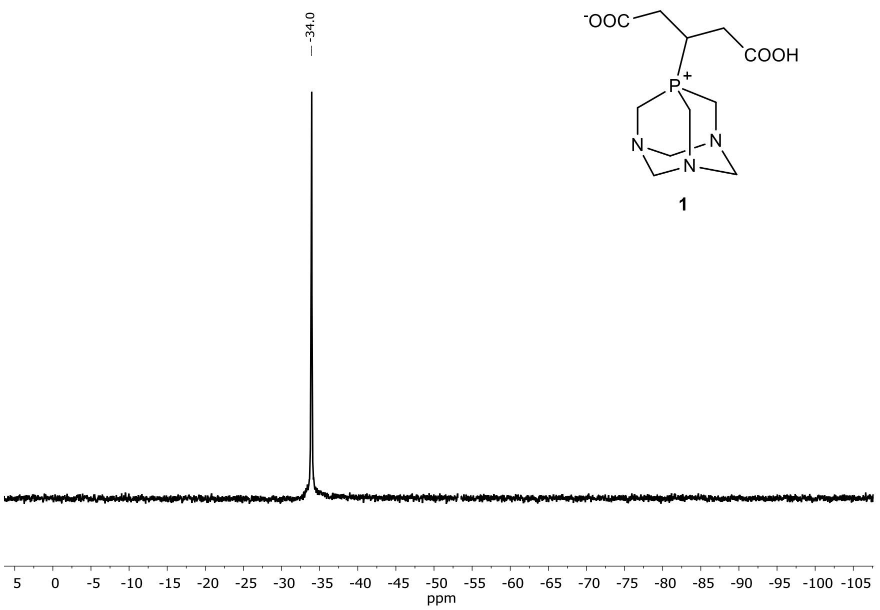

Figure S13A. ^{31}P-NMR spectrum of **1**, synthesized in a planetary ball-mill.

^{31}P{^{1}H}-NMR (145 MHz, D_2O, 25 °C): δ –33.95 (*s*) ppm.

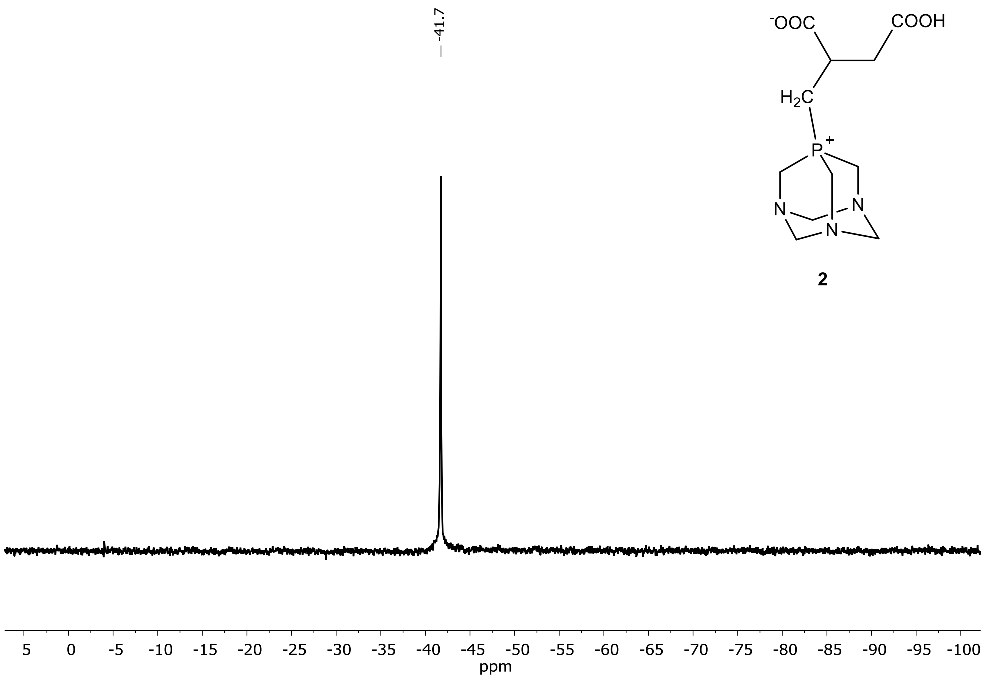

Figure S13B. ^{31}P-NMR spectrum of **2**, synthesized in a planetary ball-mill.

^{31}P{^{1}H}-NMR (145 MHz, D_2O, 25 °C): δ –41.75 (*s*) ppm.

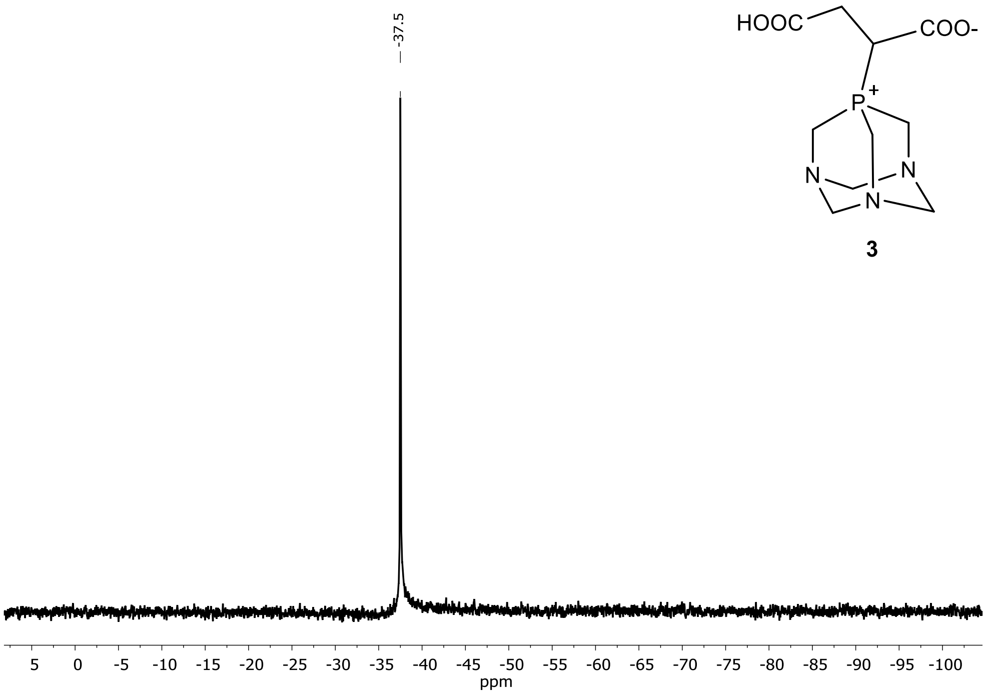

3

Figure S13C. ^{31}P-NMR spectrum of **3**, synthesized in a planetary ball-mill.

$^{31}P\{^{1}H\}$-NMR (145 MHz, D_2O, 25 °C): δ –37.5 ppm (s) ppm.

Experimental details for molecular structure determinations of phoshabetaines 1 and 2 and their coordination polymers

Diffraction measurements of **1 and 2** were taken on a Bruker-Nonius MACH3 four-circle diffractometer equipped with a point detector using graphite-monochromated Mo-Kα radiation (λ = 0.7107 Å) with the ω-scan method. Data collection was managed by CAD4 Express [S1] and XCAD4 [S2]. PSI-SCAN absorption correction was performed [S3].

Coordination polymers of **CP1.1** and **CP1.2** were measured on a Bruker Venture D8 diffractometer (INCOATEC IμS 3.0 dual CuKα and MoKα sealed tube microsources, Photon II Charge-Integrating Pixel Array detector). The data sets were collected and integrated using the APEX3 software package and MULTI-SCAN absorption correction was used [S4].

The diffraction intensity data collection of **CP2** were carried out at 293(2) K on a SuperNova diffractometer equipped with an Atlas detector using Mo Kα radiation (λ = 0.71073 Å) controlled by CrysAlisPro (Version 1.171.37.35 Agilent Technologies) [S5].

Structures were solved by the SIR-92 [S6] and SHELXT [S7] and refined by full-matrix least-squares method on $\boldsymbol{F}^2$. Non-hydrogen atoms were refined with anisotropic thermal parameters using the SHELXL package [S8] managed by WinGX [S9] and $OLEX^2$ suite [S10].

All non-hydrogen atoms were refined anisotropically. Most hydrogen atom positions were calculated geometrically and refined using the riding model, but some hydrogen atoms were refined freely. RIGU restraints were used for **2**, **CP1.2** and **CP2**.

Structures were analysed by the PLATON [S11] and publication material were prepared using the WinGX and $OLEX^2$ suites, publCIF [S12] and the Mercury program [S13].

Table S1. Crystal data and details of measurements of new phosphabetaines and their silver based CPs

	**1**	**2**	**CP1.1**	**CP1.2**	**CP2**
Chemical formula	$\mathbf{C_{11}H_{18}N_3O_4P{\times}H_2O}$	$\mathbf{C_{11}H_{18}N_3O_4P{\times}2H_2O}$	$\mathbf{C_{11}H_{18}AgN_3O_4P{\times}CF_3SO_3{\times}H_2O}$	$\mathbf{C_{12}H_{22}Ag_2F_3N_3O_7PS}$	$\mathbf{C_{24}H_{39}Ag_4F_6N_6O_{16}P_2S_2{\times}2(C_3H_6O)}$
Formula weight	305.27	323.28	562.21	656.05	1455.3
Crystal size [mm]	0.4 × 0.35 × 0.12	0.3 × 0.21 × 0.07	0.32 × 0.18 × 0.11	0.35 × 0.30 × 0.23	0.2 × 0.15×0.1
T [K]	293(2)	293(2)	295	295(15)	298(2)
λ [Å]	0.71073	0.71073	0.71073	0.71073	0.71073
Crystal system	monoclinic	triclinic	monoclinic	monoclinic	triclinic
Space group	$P2_1$/c (No. 14)	$P\bar{1}$	$P2_1/c$	$P2_1/n$	$P\bar{1}$
Crystal habit, colour	colourless, block	colourless, plate	colourless, block	colourless, block	colourless, block
a [Å]	6.954(1)	7.117(3)	13.3907(15)	13.8917(5)	7.8300(3)
b [Å]	27.496(1)	9.744(4)	10.7112(10)	10.2201(3)	15.7385(9)
c [Å]	7.7470(12)	10.281(4)	13.6383(15)	14.8851(5)	19.5242(6)
α [°]	90	87.770(10)	90	90	86.073(3)
β [°]	110.87(1)	79.100(6)	103.164(4)	104.6150(10)	89.732(3)
γ [°]	90	89.18(2)	90	90	76.740(4)
V [Å^3]	1384.1(3)	699.2(5)	1904.7(3)	2044.93(12)	2336.22(18)
Z	4	2	4	4	2
ρ_{calcd} [g cm^{-3}]	1.465	1.535	1.957	2.115	2.069
μ [mm^{-1}]	0.223	0.23	1.328	2.162	1.910
2Θ range [°]	5.82 – 51.95	5.706 – 51.95	4.886 – 52.834	4.638 –54.234	5.59 – 59.41
Index ranges	$-1 \le h \le 8$<br>$-17 \le k \le 33$<br>$-9 \le l \le 8$	$-2 \le h \le 8$<br>$-17 \le k \le 33$<br>$-9 \le l \le 8$	$-16 \le h \le 16$,<br>$-13 \le k \le 13$,<br>$-17 \le l \le 17$	$-17 \le h \le 17$,<br>$-13 \le k \le 13$,<br>$-19 \le 1 \le 18$	$-10 \le h \le 9$<br>$-21 \le k \le 19$<br>$-25 \le l \le 26$
Total reflections	3125	2787	22834	23763	20535
Unique reflections	2710[R_{int}=0.016]	2535 [R_{int}=0.027]	3900 [R_{int} = 0.0834]	4488 [R_{int} = 0.0363]	10957 [R_{int} = 0.0328,
Data/restraints/parameters	2710/4/190	2535/166/205	3900/0/269	4488/228/262	10957/549/629
Final R indices [F^2> $2\sigma(F^2)$]	0.0422	0.0946	0.0442	0.0418	0.0620
R indices (all data, w$R(F^2)$)	0.1099	0.224	0.0910	0.01317	0.1994
Goodness of fit (GOF) on F^2	1.026	1.148	1.036	1.081	1.043
$\Delta\rho_{max}/\Delta\rho_{min}$ [eÅ^{-3}]	0.26/–0.31	0.35/–0.36	0.98/–0.86	3.05/–1.03	1.70–1.26
CCDC	2038453	2038454	2038455	2038456	2038457

References in Supplementary Material

[S1] Nonius, B.V. CAD-Express Software, Ver. 5.1/1.2. Enraf Nonius, Delft, The Netherlands. 1994.

[S2] Harms, K.; Wocaldo, S. XCAD4, University of Marburg, Germany,. 1995.

[S3]North, A.C.T.; Phillips, D.C.; Mathews, F.S. A semi-empirical method of absorption correction. *Acta Cryst. A* 1968, 24, 351–359. (doi:10.1107/S0567739468000707)

[S4] Bruker (2017). APEX3 and SAINT. Bruker AXS Inc., Madison, Wisconsin, USA, 2017.

[S5] CrysAlisPro, Agilent Technologies, Version 1.171.37.35 (Release 13-08-2014 CrysAlis171 .NET) (Compiled Aug 13 2014). p CrysAlisPro, Agilent Technologies, Version 1.171.3.

[S6] Altomare, A.; Cascarano, G.; Giacovazzo, C.; Guagliardi, A. Completion and refinement of crystal structures with SIR92 *J. Appl. Crystallogr.* **1993**, *26*, 343–350. (https://doi.org/10.1107/S0021889892010331)

[S7] Sheldrick, G.M. SHELXT–Integrated space-group and crystal-structure determination. *Acta Crystallogr. Sect. Found. Adv*. **2015**, *71*, 3–8. (https://doi.org/10.1107/S2053273314026370)

[S8] Sheldrick, G.M. A short history of SHELX. *Acta Cryst. A* **2008**, *64*, 112–122. (https://doi.org/10.1107/S0108767307043930)

[S9] Farrugia, L. J. WinGX and ORTEP for Windows: an update *J. Appl. Crystallogr.* **2012**, *45*, 849–854. (https://doi.org/10.1107/10.1107/S0021889812029111 8)

[S10] Dolomanov, O.V.; Bourhis, L.J.; Gildea, R.J.; Howard, J.A.K.; Puschmann, H. OLEX^2 : A complete structure solution, refinement and analysis program. *J. Appl. Crystallogr.* **2009**, *42*, 339–341. (https://doi.org/10.1107/S0021889808042726)

[S11] Spek, A.L checkCIF validation ALERTS: what they mean and how to respond *Acta Cryst.* **2020**, *E76*, 1-11.

[S12] Westrip, S.P. publCIF: Software for editing, validating and formatting crystallographic information files. *J. Appl. Crystallogr.* **2010**, *43*, 920–925. (https://doi.org/10.1107/S0021889810022120)

[S13] Macrae, C.F.; Bruno, I.J.; Chisholm, J.A.; Edgington, P.R.; McCabe, P.; Pidcock, E.; Rodriguez-Monge, L.; Taylor, R.; Streek, J.V.D.; Wood, P.A. Mercury CSD 2.0—New features for the visualization and investigation of crystal structures. *J. Appl. Crystallogr.* **2008**, *41*, 466–470. (https://doi.org/10.1107/S0021889807067908)

[S14] Fluck, E.; Forster, J.E.; Weidlein, J.; Hadicke, E. 1.3.5-Triaza-7-phosphaadamantan (Monophospha-urotropin)/ 1,3,5-Triaza-7-phosphaadamantane (Monophospha-urotropine) *Z. Naturforsch. B Chem. Sci.* **1977**, *32*, 499–501. (https://doi.org/10.1515/znb-1977-0505)

[S15] Assmann, B.; Angermaier, K.; Paul, M.; Riede, J.; Schmidbaur, H. Synthesis of 7-Alkyl/aryl-1,3,5-triaza-7-phosphonia-adamantane Cations and Their Reductive Cleavage to Novel *N*-Methyl-*P*-alkyl/aryl[3.3.1]bicyclononane Ligands. *Chem. Ber.* **1995,** *128*, 891–900. (https://doi.org/10.1002/cber.19951280907)

[S16] Tang, X.; Zhang, B.; He, Z.; Gao, R.; He, Z. 1,3,5-Triaza-7-phosphaadamantane (PTA): A Practical and Versatile Nucleophilic Phosphine Organocatalyst. *Adv. Synth. Catal.* **2007**, *349*, 2007–2017. (https://doi.org/10.1002/adsc.200700071)

[S17] Udvardy, A.; Purgel, M.; Szarvas, T.; Joó, F.; Kathó, Á. Synthesis and structure of stable water-soluble phosphonium alkanoate zwitterions derived from 1,3,5-triaza-7-phosphaadamantane. *Struct. Chem.* **2015**, *26*, 1323–1334. (https://doi.org/10.1007/s11224-015-0618-4)

Table S2. Selected bond lengths and angles of PTA and its derivatives

	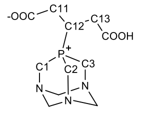	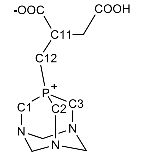	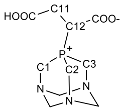	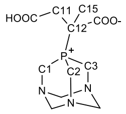	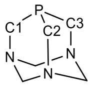	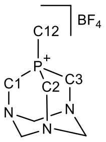	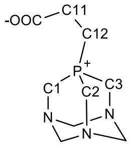
	**1** [this work]	**2** [this work]	**3**, AHISOB **[S17]**	AHISUH **[S17]**	TAZPAD, PTA **[S14]**	MTZPAD **[S15]**	SIJPOR **[S16]**
Bond distances (Å)							
C1–P	1.823(3)	1.788(7)	1.819(9)	1.822(8)	1.856(5)	1.812(3)	1.832(2)
C2–P	1.827(2)	1.806(6)	1.810(9)	1.789(8)	1.856(4)	1.811(3)	1.831(2)
C3–P	1.825(3)	1.796(7)	1.819(9)	1.811(8)	1.856(5)	1.808(3)	1.824(2)
Cl2–P	1.823(2)	1.778(6)	1.826(8)	1.818(9)	N/A	1.772(2)	1.807(2)
C11–C12 and C12–C13	1.530(4) 1.532(3)	1.526(8)	1.499(10)	1.508(12)	N/A	N/A	1.518(2)
Bond angles (°)							
C1–P–C2	103.26(11)	102.8(3)	101.6(4)	100.0(4)	96.06	101.5	99.6(9)
C1–P–C3	100.99(12)	100.3(3)	102.3(4)	100.9(4)	96.06	101.8	100.1(9)
C2–P–C3	100.39(12)	101.3(3)	100.4(4)	102.2(4)	96.06	103.2	102.4(9)
C1–P–C12	120.74(11)	121.7(3)	113.8(4)	113.2(4)	N/A	115.9	121.2(9)
C2–P–C12	120.54(10)	118.7(3)	112.6(4)	115.1(4)	N/A	115.0	105.9(9)
C3–P–C12	107.43(11)	108.8(3)	123.3(4)	122.3(4)	N/A	117.3	121.2(9)

N/A – not applicable

Structural characterization of the phosphabetaines 1 and 2

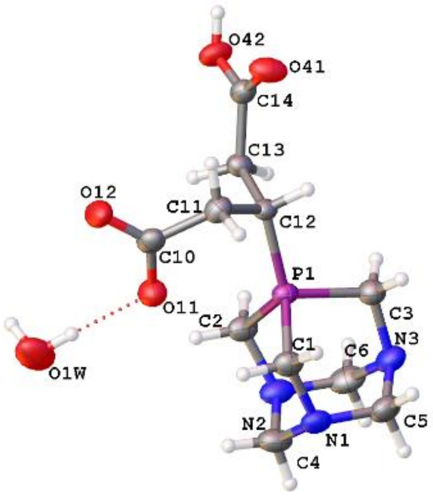

Figure S14. ORTEP diagram of the asymmetric unit of **1**×H_2O showing the atom labelling scheme. (Thermal ellipsoids are shown at a 50% probability level.

Table S3. Selected hydrogen bonds (including weak C–H…O interactions) in **1**

D—H···*A*	*D*—H	H···*A*	*D*···*A*	*D*—H···*A*
O1W – H1WA .. O11	0.86(3)	1.92(3)	2.781(3)	176.8(19)
O1W – H1WB .. N1$^{(i)}$	0.855(15)	2.075(18)	2.889(3)	159(3)
O42 – H42 .. O12$^{(ii)}$	0.85(2)	1.72(2)	2.571(3)	180(4)
C1 – H1A .. O41$^{(ii)}$	0.9700	2.4800	3.242(3)	136.00
C1 – H1B .. O11	0.9700	2.5400	3.078(3)	115.00
C2 – H2A .. O11	0.9700	2.3800	2.954(3)	118.00
C2 – H2B .. O1W$^{(iii)}$	0.9700	2.3600	3.234(4)	150.00
C3 – H3A .. O11$^{(iii)}$	0.9700	2.5000	3.439(4)	162.00
C6 – H6A .. O1W$^{(iii)}$	0.9700	2.4800	3.353(4)	150.00
C11 – H11B .. O41	0.9700	2.5500	3.121(3)	118.0

Symmety codes: (i) x,y,–1+z; (ii) 1–x,–y,–z; (iii) –1+x,y,z

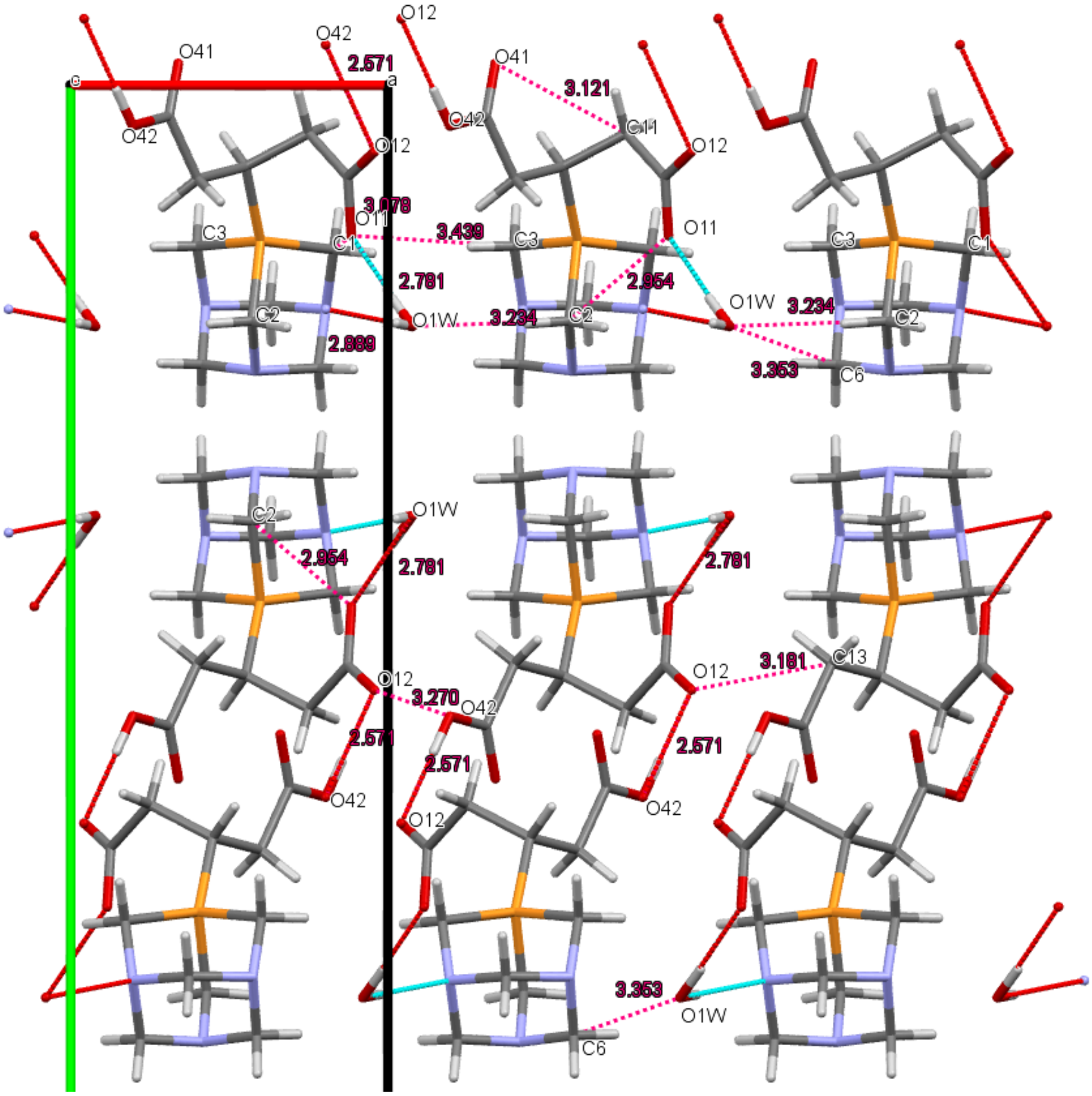

Figure S15. Partial packing diagram of **1** along axis „**c**” with selected hydogen bonds

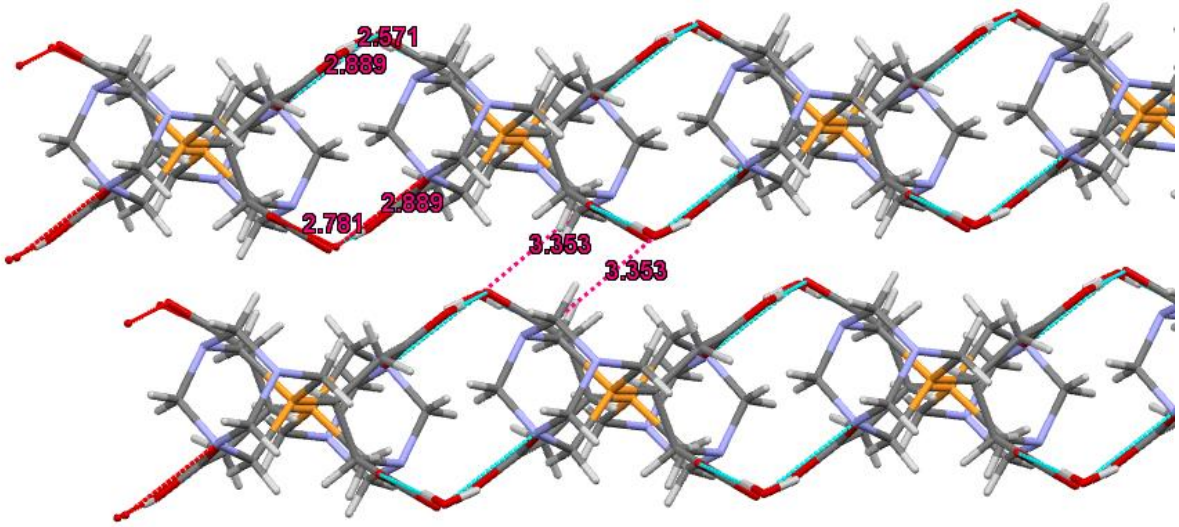

Figure S16. Partial packing view of **1** (chains)

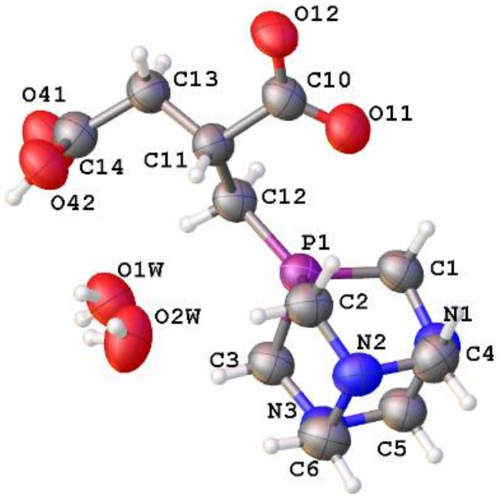

Figure S17. ORTEP diagram of the asymmetric unit of **2**×2 H_2O showing the atom labelling scheme. (Thermal ellipsoids are shown at a 50% probability level.

Table S4. Hydrogen bonds (including weak C–H…O interactions) in **2**

D—H···A	D—H	H···A	D···A	D—H···A
O1W – H1WA .. O41$^{(i)}$	0.86(5)	2.10(5)	2.934(7)	164(4)
O1W – H1WB .. O11$^{(ii)}$	0.85(5)	2.28(5)	3.046(7)	151(6)
O2W – H2WA .. O11$^{(ii)}$	0.84(5)	1.89(5)	2.725(8)	172(5)
O2W – H2WB .. O12$^{(iii)}$	0.84(4)	2.00(4)	2.806(8)	162(7)
O42 – H42 .. O12$^{(ii)}$	0.85(5)	1.73(5)	2.515(7)	153(7)
C1 – H1A .. O41$^{(iii)}$	0.9700	2.4400	3.229(9)	138.00
C1 – H1B .. O11	0.9700	2.4100	2.981(8)	117.00 .
C2 – H2B .. O2W	0.9700	2.3900	3.202(9)	141.00 .
C3 – H3A .. O2W	0.9700	2.5200	3.299(9)	138.00 .
C12 – H12B .. O1W	0.9700	2.5400	3.434(8)	153.00 .

Symmety codes: (i) 3–x,1–y,1–z; (ii) 1+x,y,z; (iii) 2-x,1–y, 1–z

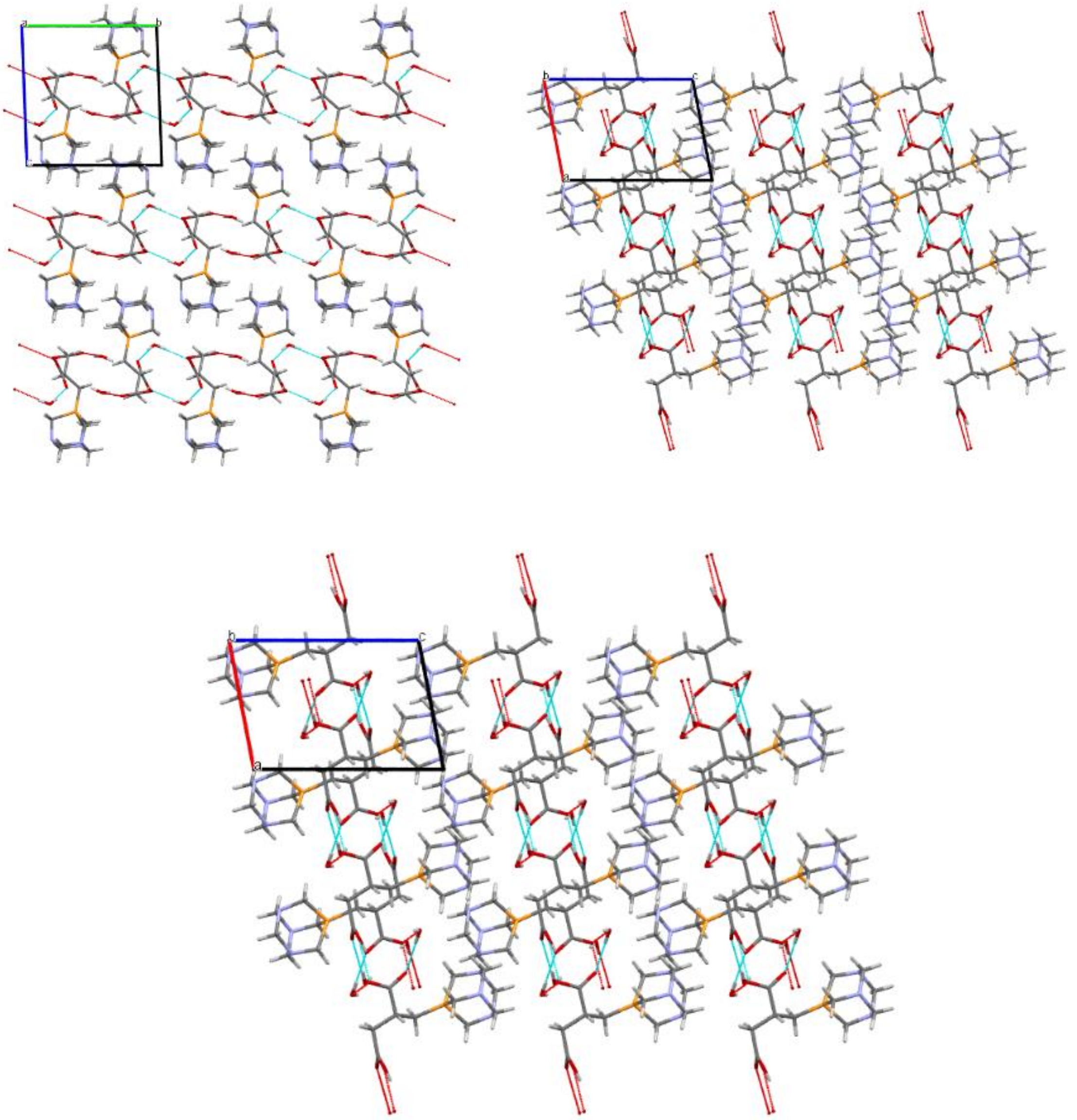

Figure S18. Packing diagrams of **2** along the axes „**a**”, „**b**”, and „**c**”.

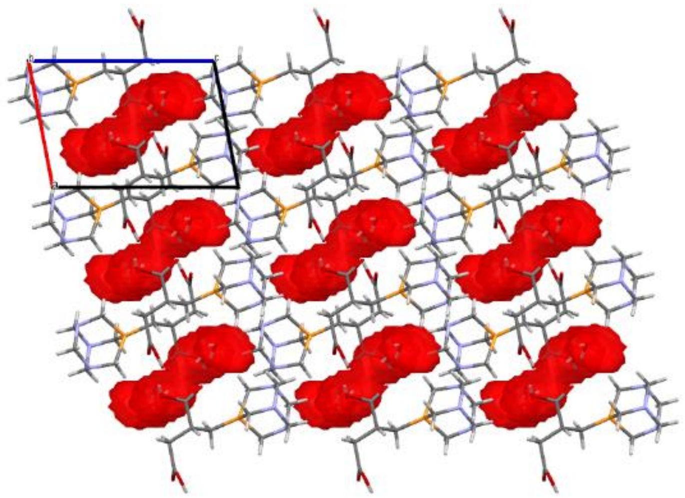

Figure S19. Water molecules in **2** along axis „**c**”

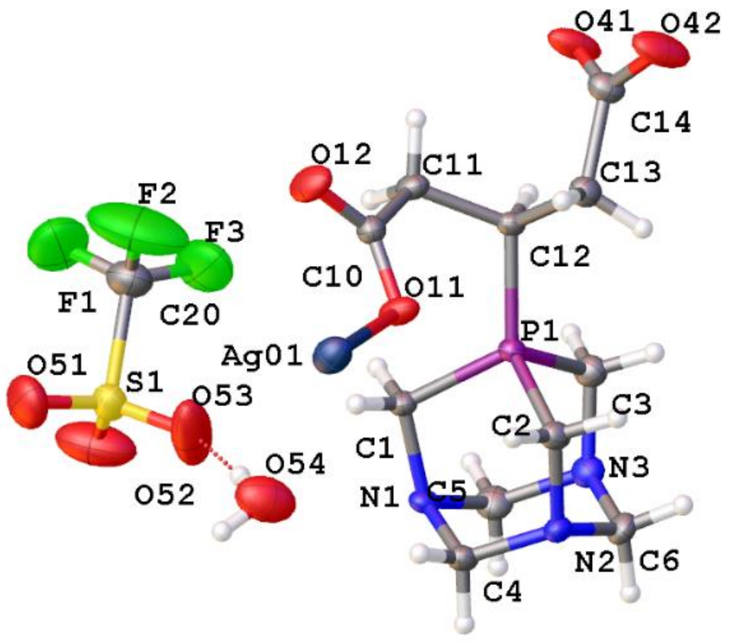

Figure S20. ORTEP diagram of the asymmetric unit of **CP1.1** showing the atom labelling scheme. (Thermal ellipsoids are shown at a 50% probability level.

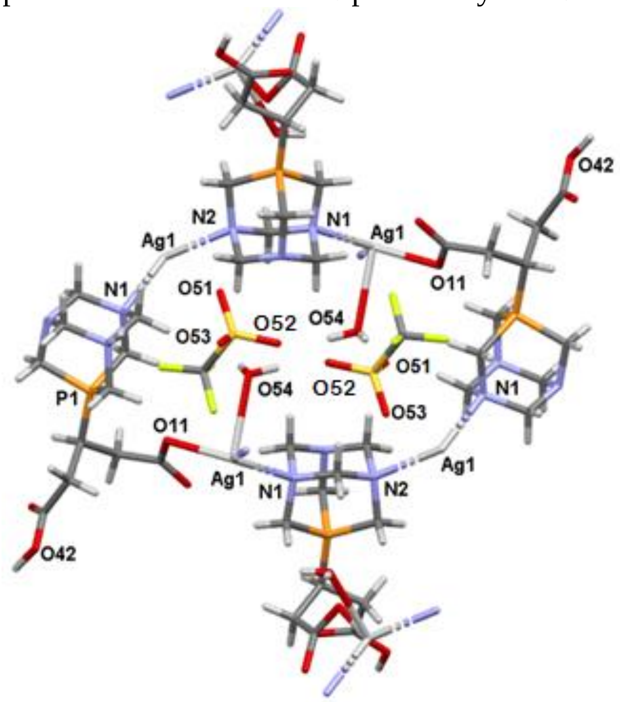

Figure S21. Partial packing view of **CP1.1**

Table S5. Hydrogen bonds (including weak C–H…O interactions) in **CP1.1**

D—H···*A*	*D*—H	H···*A*	*D*···*A*	*D*—H···*A*
O42 – H42 .. O12$^{(i)}$	1.03(8)	1.57(8)	2.583(5)	172(7)
O54 – H54A .. O53	0.8500	2.0000	2.840(6)	173.00
O54 – H54B .. O51$^{(ii)}$	0.8500	2.1800	2.893(6)	142.00
C1 – H1A .. O11	0.9700	2.5500	3.104(5)	116.00
C1 – H1A .. O53	0.9700	2.3900	3.154(6)	136.00
C1 – H1B .. O41$^{(iii)}$	0.9700	2.4100	3.170(5)	135.00
C1 – H1B .. O12$^{(iv)}$	0.9700	2.5000	3.303(5)	140.00
C2 – H2A .. O51$^{(v)}$	0.9700	2.3600	3.283(5)	159.00
C2 – H2A .. O54$^{(vi)}$	0.9700	2.5400	3.177(6)	123.00
C2 – H2B .. O11	0.9700	2.4300	3.005(5)	118.00
C4 – H4A .. O52$^{(vii)}$	0.9700	2.6000	3.355(7)	135.00
C5 – H5B .. O12$^{(iv)}$	0.9700	2.5900	3.369(6)	137.00
C11 – H11A .. O41	0.9700	2.5600	3.097(5)	115.00
C11 – H11B .. O41$^{(iii)}$	0.9700	2.5500	3.205(6)	125.00
C12 – H12 .. O41	0.9800	2.3300	2.706(5)	102.00
C12 – H12 .. O41$^{(iii)}$	0.9800	2.5100	3.083(5)	117.00

Symmetry codes: (i)1-x,–1/2+y,3/2–z; (ii) –x,2–y,1–z; (iii)1–x,1–y,1–z; (iv) x,3/2–y,–1/2+z; (v) x,–1+y,z; (vi) –x,1–y,1–z; (vii) –x,–1/2+y,1/2–z

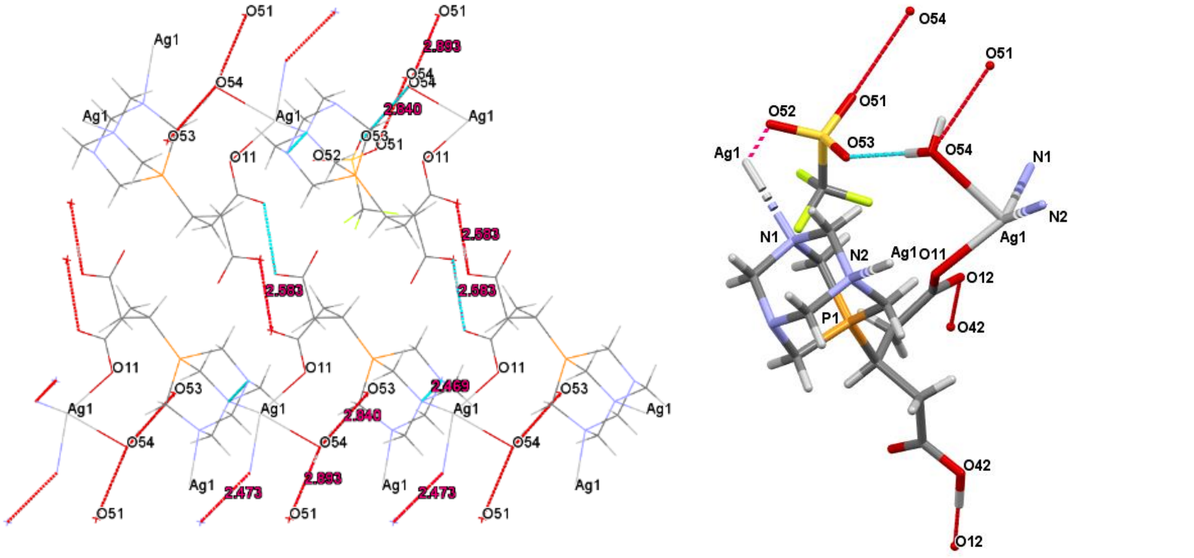

Figure S22. Packing diagrams of **CP1.1** with strong hydrogen bonds (left) and geometry of silver ion (right).

Selected bond lenghts: Ag1–O11=2.294(3), Ag1–N2$^{(i)}$=2.439(3), Ag1–N1$^{(ii)}$=2.465(3), Ag1–O54=2.691(4), P1-O11=2.798(3), P1–C12=1.818(4), Ag1–O12=2.963(3), Ag1–O52$^{(iii)}$=3.166(6) O42–H42...O12$^{(iv)}$=2.583(5), O12–H12...O41=2.706(5), O54–H54A...O53=2.840(6), O54–H54B...O51$^{(v)}$=2.893(6), weak interactions: Ag1–O53$^{(iii)}$=3.248(6), [Symmetry codes: (i) –x,1-y,1–z, (ii) x,3/2–y,1/2+z (iii) x,3/2–y,1/2+z (iv) 1–x,-1/2+y,3/2–z, (v) –x,2–y,1–z).

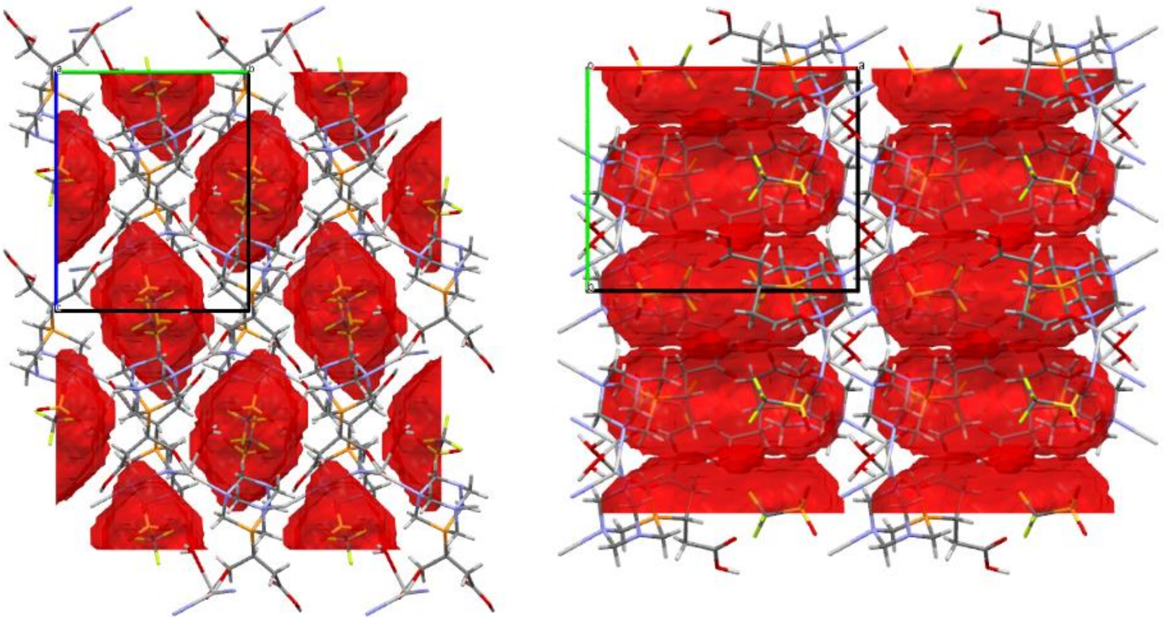

Figure S23. Triflate anions in **CP1.1** along axes „**a**” and **„c”**

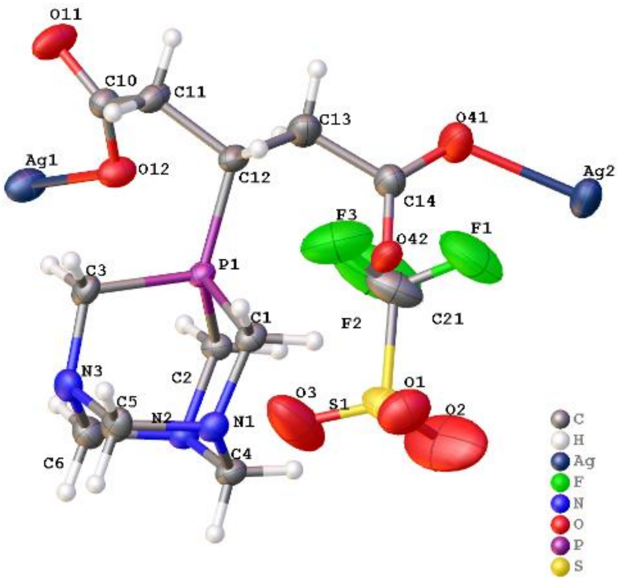

Figure S24. ORTEP diagram of the asymmetric unit of **CP1.2** showing the atom labelling scheme. (Thermal ellipsoids are shown at a 50% probability level.

Table S6. Hydrogen bonds (including weak C–H…O interactions) in **CP1.2**

D—H···*A*	*D*—H	H···*A*	*D*···*A*	*D*—H···*A*
C1 – H1A .. O42	0.9700	2.3500	2.980(5)	122.00
C1 – H1A .. O11$^{(i)}$	0.9700	2.4500	3.259(6)	141.00
C1 – H1B .. O1$^{(ii)}$	0.9700	2.1500	3.114(8)	171.00
C2 – H2A .. O12	0.9700	2.5500	3.125(5)	118.00
C2 – H2B .. O2	0.9700	2.3800	3.287(7)	155.00
C2 – H2B .. O42	0.9700	2.5100	3.100(5)	119.00
C3 – H3B .. O12	0.9700	2.4500	3.052(5)	120.00
C4 – H4A .. O11$^{(i)}$	0.9700	2.4800	3.274(6)	139.00
C4 – H4B .. O1$^{(iii)}$	0.9700	2.6000	3.565(10)	174.00
C5 – H5A .. O2$^{(iii)}$	0.9700	2.5100	3.405(6)	153.00
C6 – H6A .. O3$^{(iv)}$	0.9700	2.5000	3.374(7)	151.00
C11 – H11B .. O41$^{(v)}$	0.9700	2.5100	3.310(6)	140.00
C13 – H13A .. O12	0.9700	2.5600	3.131(6)	117.00

Symmetry codes: (i) –1/2+x,3/2–y,–1/2+z (ii) x,1+y,z (iii) 1/2–x,1/2+y,1/2–z (iv) 1–x,1–y,1–z (v) 1/2–x,1/2+y,3/2–z

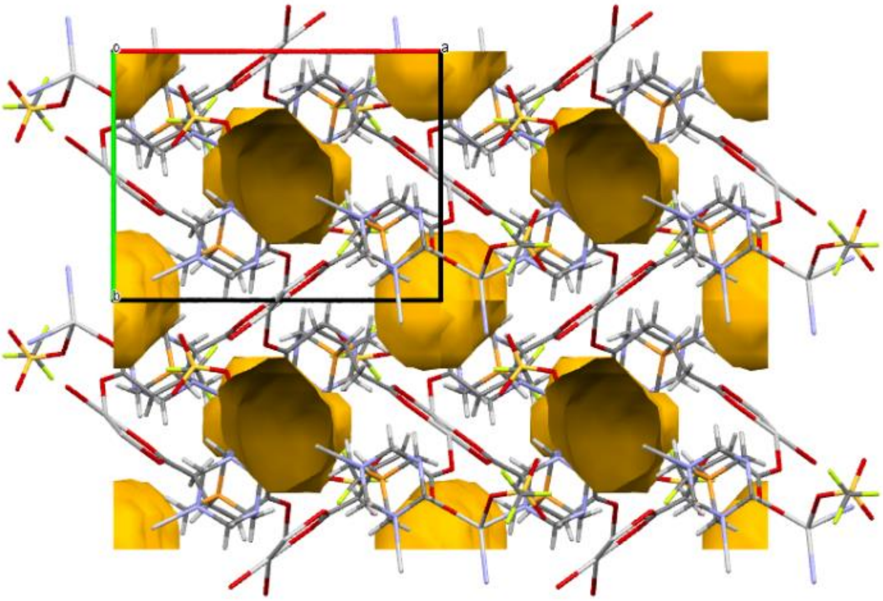

Figure S25. Voids in **CP1.2**

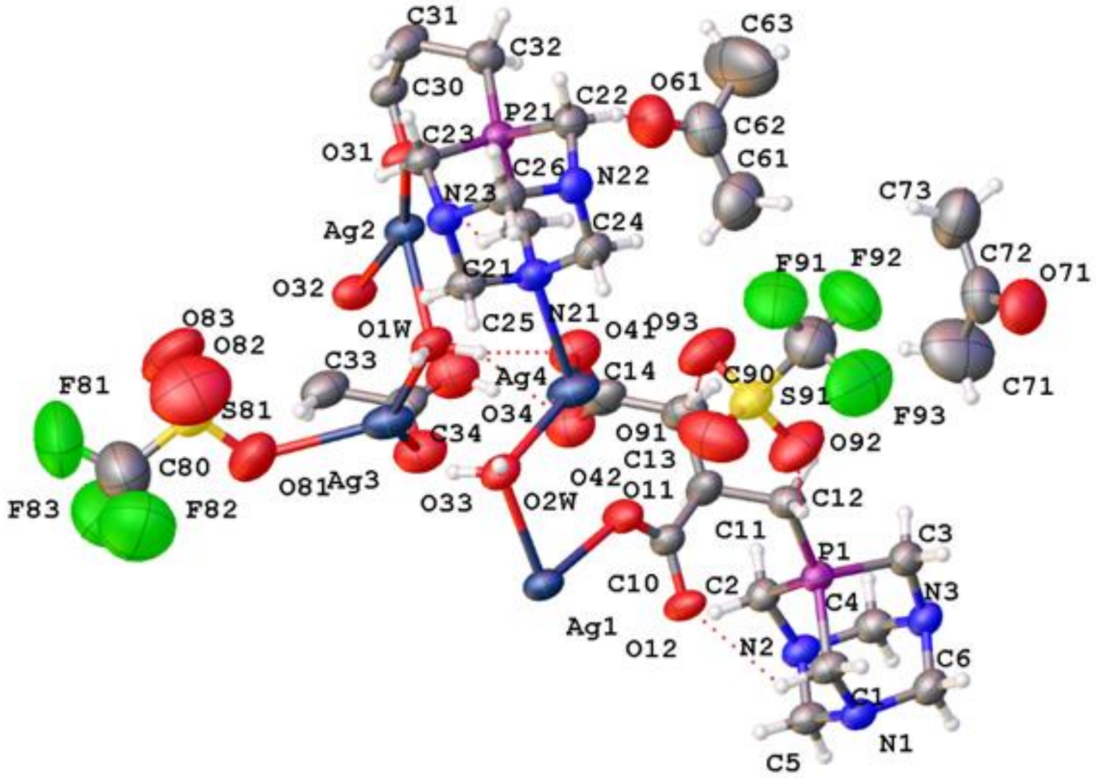

Figure S26. ORTEP diagram of the asymmetric unit of **CP2** showing the atom labelling scheme. (Thermal ellipsoids are shown at a 50% probability level.

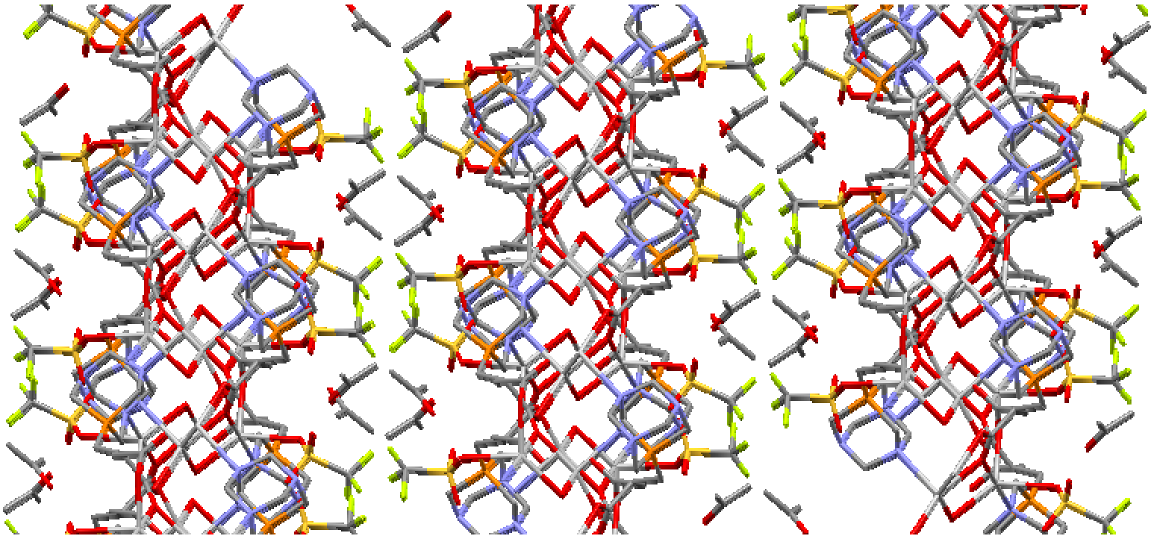

Figure S27. Partial view of the crystal lattice of **CP2** showing the channels of acetone

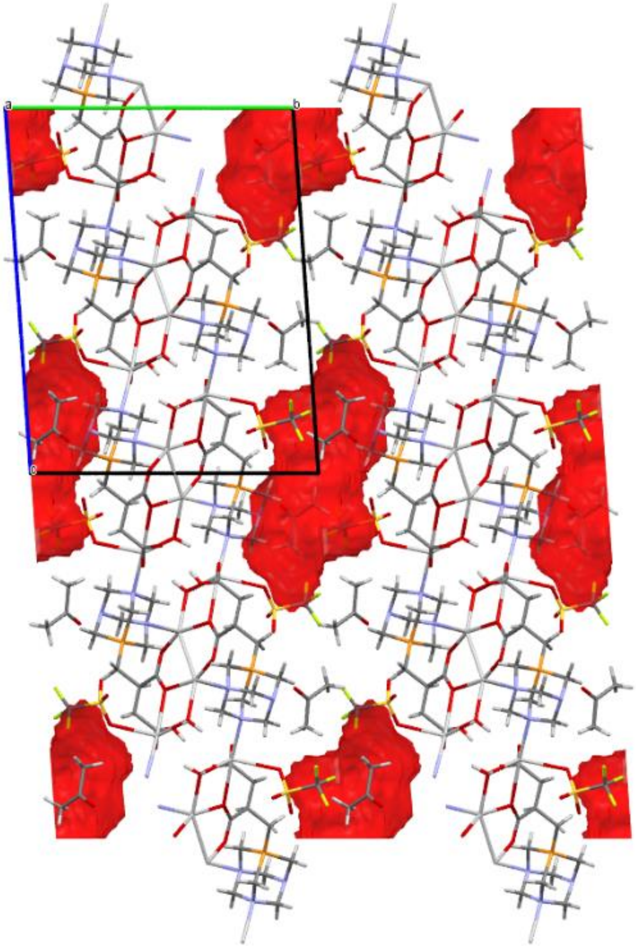

Figure S28. Triflate anions in CP2 along axis „**a**”
